# Development, hotspots, and trend directions of research on green production behavior of farmers based on CiteSpace and VOSviewer visual analysis

**DOI:** 10.3389/fnut.2025.1604525

**Published:** 2025-07-02

**Authors:** Bin Zhang, Wenyi Mao, Zhen Hu

**Affiliations:** ^1^School of Economics and Management, Jiangxi Agricultural University, Nanchang, Jiangxi, China; ^2^School of Humanities and Public Administration, Jiangxi Agricultural University, Nanchang, Jiangxi, China

**Keywords:** farmers, green production behavior, bibliometric analysis, CiteSpace, VOSviewer

## Abstract

**Introduction:**

Promoting the popularization of green production behavior among farmers is the key to achieving sustainable agricultural development, guaranteeing food security and protecting the ecological environment.

**Methods:**

Based on the Web of Science core database, this study used CiteSpace and VOSviewer to conduct a bibliometric analysis of 697 literatures on green production behaviors of farmers from 2000 to 2024, aiming to sort out the development history and characteristics of this research field.

**Results and discussion:**

The results show that the number of literature in this field continues to grow, and the studies are concentrated in the field of environmental sciences, and most of them are published in Sustainability journals; China is the largest contributor to the literature in this field, and it cooperates closely with the United States, and the research institutes and authors are mainly from the Chinese institutions of higher education; the studies are mainly focused on the three dimensions of the behavioral drivers, the development of the theoretical models, and the evaluation of the benefits of the implementation. The current research hotspots include behavioral implementation benefits, influencing factors of behavioral adoption, green production methods, and the impact of green revolution on behavioral adoption. This study systematically summarizes the research hotspots, challenges and future development directions in the field of farmers' green production behaviors, and provides an important reference for subsequent theoretical research and innovative practices.

## 1 Introduction

In recent years, the increasing severity of environmental issues and the evolving concept of sustainable development have made green production practices essential to agricultural development. The Global Environment Outlook report highlights that the impact of agricultural production activities on the environment has become increasingly significant, leading to problems such as soil degradation, water resource pollution, and loss of biodiversity (1). Consequently, there is an urgent need for a green transformation of agricultural production methods to address these challenges. As a crucial component of human food security and ecological balance, the greening of agricultural production methods is vital for achieving sustainable agricultural development (2). As the main body of agricultural production and the key actor of environmental protection, the implementation of green production behavior by farmers is an important measure to deal with agricultural environmental pollution and ensure food security. Research indicates that the green production behaviors adopted by farmers can mitigate the negative impacts of climate change on agricultural production to varying degrees, while also reducing environmental risks and economic losses during the production and operational processes (3). Furthermore, through green production, farmers can enhance the quality and market competitiveness of agricultural products, decrease the use of chemical fertilizers and pesticides, improve resource use efficiency, lower production costs, and promote the sustainability of agriculture (4). Therefore, studying the green production behaviors of farmers is of great significance for advancing global environmental governance and ensuring food security.

Currently, the meaning of green production behavior is deeply explored in the academic community. While green production encompasses various interpretations, its core focus remains on the greening of the production process. Shao Xuemin, Chairman of the Thematic Committee on Environment and Energy of the United Nations in China, suggested at the World Productivity Conference that production efficiency and green productivity fundamentally represent cleaner production (5). Scholars such as Khalii view green production as a model of sustainable development, which includes the operational aspects of enterprises, environmental sustainability, and the minimization, recycling, and reuse of waste (6). In addition, in 2019, the United Nations Environment Programme specified that green production is a strategy for controlling pollution and using resources efficiently throughout the production process, aiming to maximize resource efficiency and minimize environmental impacts through technological innovations in order to promote coordinated ecological and economic development, and that it plays an increasingly critical role in the industrial sector and environmental protection (7). On the basis of the above concepts, some scholars apply it in the field of agriculture, interpreting it as the ecological and environmental protection behaviors of farmers in the process of agricultural production in a green way, such as “reducing the amount of chemical fertilizers and pesticides”, “scientific use of no-tillage technology”, “recycling agricultural waste” and so on (8). The United Nations Environment Programme (UNEP) defines green production behavior as a production method that not only ensures and increases agricultural productivity and profitability, but also reduces pollution of the rural environment and improves the efficiency of resource use (9). Based on the definition of green production behaviors, green production behaviors in agriculture cover no-tillage or less-tillage techniques, crop rotation, crop sets and intercropping systems, organic fertilizers and soil test formula fertilization techniques, the use of bio pesticides, straw return techniques, and biogas utilization of wastes (10). These behaviors cover the entire process of agricultural production, including pre-production, production and post-production (11).

Focusing on the connotation of green production, scholars have used inductive summarization method and literature analysis method to review and explore the green production behavior, which mainly involves the relationship between agricultural socialization services and farmers' green production (12), the factors influencing the green production behavior, the motivation, the research method and other dimensions (67). However, although these review articles provide a certain reference value in the induction and generalization of literature materials, most of them stay in the qualitative analysis of the research status, lack of discussion of in-depth content such as the co-citation relationship between the literature, the evolution of the theme context, etc., and the analysis of the basic characteristics and attributes of the knowledge contained in the literature is slightly insufficient. In addition, the existing literature relies more on subjective cognition and lacks the necessary objectivity in organizing the current status of research. However, modern bibliometric software such as CiteSpace, VOSviewer, etc., which use mathematical and statistical models to quantitatively analyze the literature and quantitatively process the literature, authors, and other information, have significant advantages in processing large-scale literature, and provide a certain scientific basis for sorting out the research results in this field (13).

With the continuous advancement of computer engineering techniques, bibliometric analysis is increasingly being utilized to assess literature reviews. First proposed by Alan Pritchard in 1969, this methodology effectively supports in-depth analysis and reviews of progress on topics covered by various journals, institutions, countries, and authors (14). It relies on quantitative studies of a substantial number of publications and aims to provide a qualitative description of research trends. Consequently, researchers can more easily discover and evaluate the dynamics of academic research by identifying key influential articles. For instance, literature on studies related to farm household behavior, such as farmers' climate adaptive behavior (15) and livelihood strategy choices (16), has been analyzed through bibliometric. However, there has yet to be a bibliometric analysis focusing on the green production behavior of farm households.

In view of this, this study intends to conduct a systematic compilation and in-depth bibliometric analysis of the literature related to green production behavior of farmers during the period of 2000–2024 based on the Web of Science Core Collection database, using CiteSpace and VOSviewer software. This study will visualize and analyze the overall output, research subjects and cooperative networks, highly cited literature, keyword co-occurrence and cluster analysis, aiming to comprehensively explore the research hotspots and future development trends in this field, and deeply analyze the gaps and deficiencies in the current research, so as to provide useful reference for the subsequent deepening of the research on green production behavior of agricultural households. This study aims to provide scholars, researchers and policy makers with accurate and systematic descriptive information about the literature on green production behavior of farmers, so as to contribute to food security and sustainable development of agriculture.

This study focuses on the following key questions: first, how has the research dynamics in the field of green production behavior of farmers evolved over the past two decades? What are the academic journals and subject areas relevant to the field? Second, what is the distribution of research on green production behavior of farmers at the national, institutional and author levels? Third, what is the knowledge base and seminal literature in the field? What is the trend of progress in the research theme? Fourth, what are the current hot topics and their evolutionary trajectories in the study of green production behavior of farmers? What is the contribution of related research to academic quality?

## 2 Materials and research methods

### 2.1 Research methodology

CiteSpace is visualization software developed by Prof. Chaomei Chen's team based on the Java platform, which mines literature data and performs cluster analysis and other visualization software (17). The software is able to explore the intellectual background and current cutting-edge topics of a specific field of research, and detect the evolution of disciplines and fields through the analysis of literature co-citation, collaborative networks, and contributions to themes and fields (18). By combing the literature systematically through quantitative and visualization, the research results, collaboration networks, and research hotspots of a specific discipline within a certain time span can be visualized in the form of graphs (19). In this study, CiteSpace 6.4. R1 software is used to analyze the research hotspots and frontier dynamics in the field of agricultural green production behaviors from the perspective of bibliometric. The time slice is set from 2000-01 to 2024-12, and the width of the time slice is set to 1 year. The node type will be selected according to the content of this study. The top N is 50, and the N% thresholds are set to 10%, 100. Other options are set by default. In general, in CiteSpace visualization mapping, nodes with high centrality are usually connected to many other nodes, indicating that the content of the node is a core or important concept in the research field. Meanwhile, the strength of connections between nodes implies that they are interconnected in that research area. In addition, cluster analysis is used by CiteSpace to identify and visualize different subfields or topics in a research area, where clusters are numbered to indicate their size, with #0 denoting the largest cluster containing the most nodes, #1 denoting the second largest cluster, and so on.

VOSviewer is a visual knowledge mapping software developed by van Eck and Waltman from the Center for Science and Technology Research at Leiden University in the Netherlands, in 2009 (20). VOSviewer boasts robust visualization capabilities, making it suitable for large-scale datasets. It supports four browsing modes: labeled view, density view, clustering view, and dispersion view. In this study, we utilized VOSviewer to visualize and analyze keywords, with the aim of revealing research dynamics and development trends in this subject area.

Although both Software have their unique strengths, combining them can effectively establish a network of connections between knowledge units in the literature and clearly illustrate the overall structure of the knowledge domain. For example, VOSviewer is good at working with large data sets and is easy to operate, while CiteSpace is good at finding internal connections within the data and analyzing specific topics. The integration of these two tools can provide a more comprehensive coverage of research data and enhance research efficiency (21). Therefore, this study will utilize CiteSpace 6.4.R1 and VOSviewer 1.6.20 to investigate the current status and trends in research on the green production behaviors of farmers through a comprehensive application of both quantitative and qualitative methods in the literature.

### 2.2 Data sources

**Figure 1:**
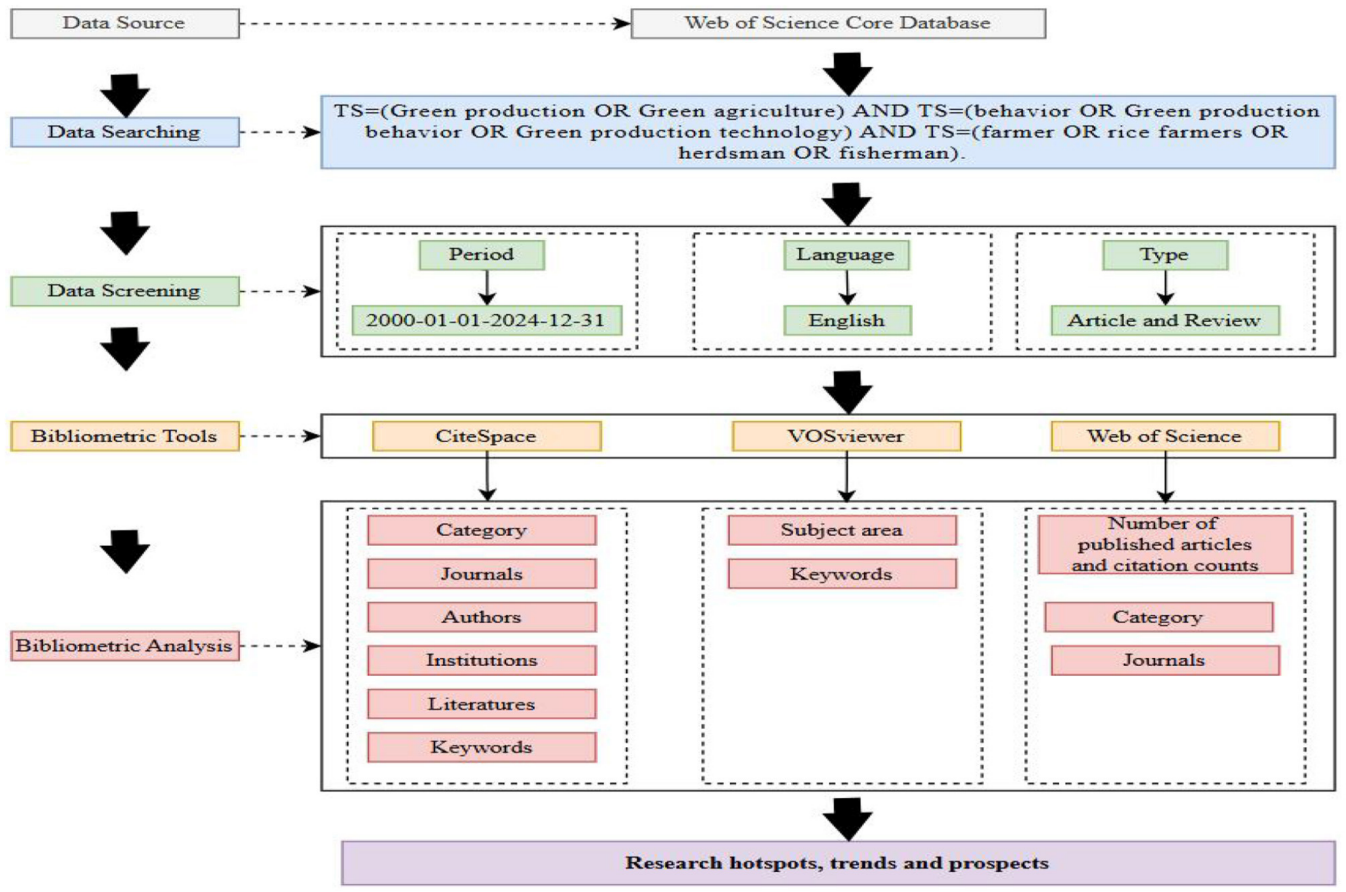
Research workflow chart.

The literature data for this study is derived from the Web of Science (WOS) Core Collection, a database of authoritative and highly influential journal articles from around the world. It is one of the most widely recognized and important data sources in the field of bibliometric analysis. The literature search was conducted simultaneously and independently by three researchers on January 1, 2025, and was completed within one day to avoid potential biases that could be introduced by data updates and to ensure the accuracy of the collected data. The search timeframe was set from January 1, 2000, to December 31, 2024. An accurate and appropriate search formula is essential for comprehensively and precisely collecting literature in a specific academic field. The WOS Core Collection was searched using subject terms (TS), which were combined with in the following search format: TS = (Green production OR Green agriculture) AND TS = (behavior OR Green production behavior OR Green production technology) AND TS = (farmer OR rice farmers OR herdsman OR fisherman). The scope of search is “Web of Science core database”, the category of literature is set as “ARTICLE”, “Review ARTICLE”, and the language is “ENGLISH”. After a manual screening process, literature that did not conform to the research topic was systematically removed, resulting in a final total of 697 valid articles, which were exported in plain text format. These documents contain detailed information, including the country, author, author's affiliation, and year of publication, which constitute the basic dataset analyzed in this study. The retrieved literature was downloaded and imported into CiteSpace software, where the data was processed using the deduplication function to remove duplicates. Subsequently, the collected data was visualized in both CiteSpace and VOSviewer. The entire retrieval and analysis process is illustrated in Figure 1.

### 2.3 Data pre-processing

**Figure 2:**
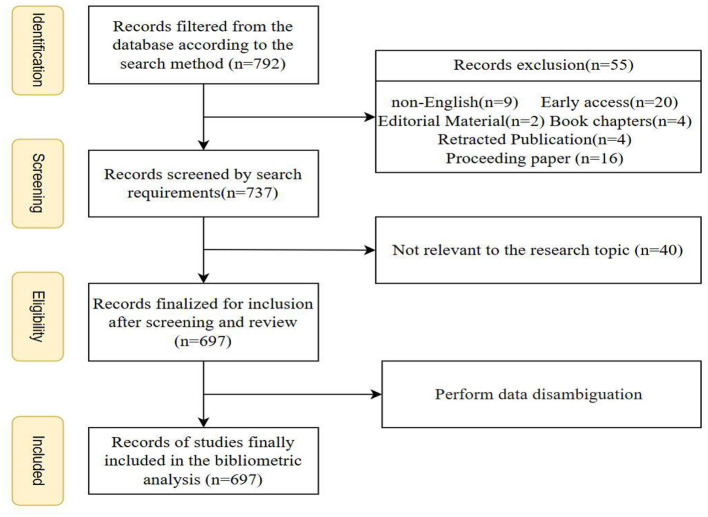
PRISMA selection process flowchart.

First, 792 documents were screened from the database based on the search method. Next, these were further screened to exclude 55 non-compliant documents, including non-English documents (9), early access documents (20), editorial materials (2), book chapters (4), withdrawn publications (4), and conference papers (16). This step was taken to ensure data quality and relevance of the analysis. Then, 737 documents were screened according to the search requirements, and 40 documents that were not relevant to the study topic were further excluded. After screening and reviewing, 697 documents were finally identified for the bibliometric analysis. This step was taken to ensure that all the literature included in the analysis was closely related to the study topic and was of high quality after rigorous screening and review. Finally, data de-weighting was performed to ensure the accuracy of the analysis and to avoid double counting. The entire data processing flow is detailed in Figure 2.

In addition, in order to ensure the accuracy and consistency of the data, this study also standardized the names of the institutions, such as unifying different forms of the name into a consistent name, e.g., “The Chinese University of Hong Kong” and “The University of Hong Kong” were standardized into a consistent name. The keyword data were processed by combining synonyms (e.g. “GPB” and “green production practices”) and standardizing plural forms (e.g., “farmer” and “farmers”).

Export the retrieved documents in “Refwork” format, rename the file as “download_WOS_txt”, create new input and output folders, and convert them to formats that can be recognized and processed by CiteSpace software; at the same time, use CiteSpace software to convert them to WOS-related formats to ensure that VOSviewer software can recognize them. At the same time, CiteSpace software was used to convert the file into WOS-related formats to ensure that VOSviewer software could recognize the format.

Using Excel software to record, sort and filter the overall output, time distribution characteristics and trends of literature, frequency of cited literature, etc., and using CiteSpace and VOSviewer software to visualize and analyze the literature data obtained above, visualize and analyze the authors, institutions, keywords and other types of nodes, and draw the corresponding knowledge maps to mine the knowledge of farmers. Green Production Behavior research hotspots and research directions.

## 3 Bibliometric results and analysis

### 3.1 Distribution power

#### 3.1.1 Descriptive statistical analysis of the literature

Table 1 presents detailed information regarding the literature on the green production behavior of farmers from 2000 to 2024. After removing duplicates, a total of 697 valid publications were identified. This study integrates the research results of 2,585 scholars from 1,649 research institutions in 172 countries around the world, distributed in 281 different academic journals. As of the data retrieval cut-off date, these articles had garnered 17,573 citations, resulting in an average annual citation rate of 702.92 and an average of 25.21 citations per paper. H-index (H-index) is an important index for measuring the influence of scientific research, which refers to a certain period of time in which the citation frequency of H articles published is not less than H times, and the higher the H-index is, the stronger the academic influence in the field is, and the research results are widely cited with high academic reputation, which is identified as 66 in this study (22).

**Table 1:** Descriptive characteristics of literature on green production behavior of farmers.

**Description**	**Results**	**Description**	**Results**
Timespan	2000–2024	Author's keywords	2,570
Articles	697	Keywords plus	1,693
Journals	281	Citing articles	14,873
Authors	2,585	Citations	17,573
Institutions	1,649	Average citations per year	702.92
Countries	172	Average per item	25.21
References	36,331	H-index	66

#### 3.1.2 Trends in publications and disciplinary distribution

**Figure 3:**
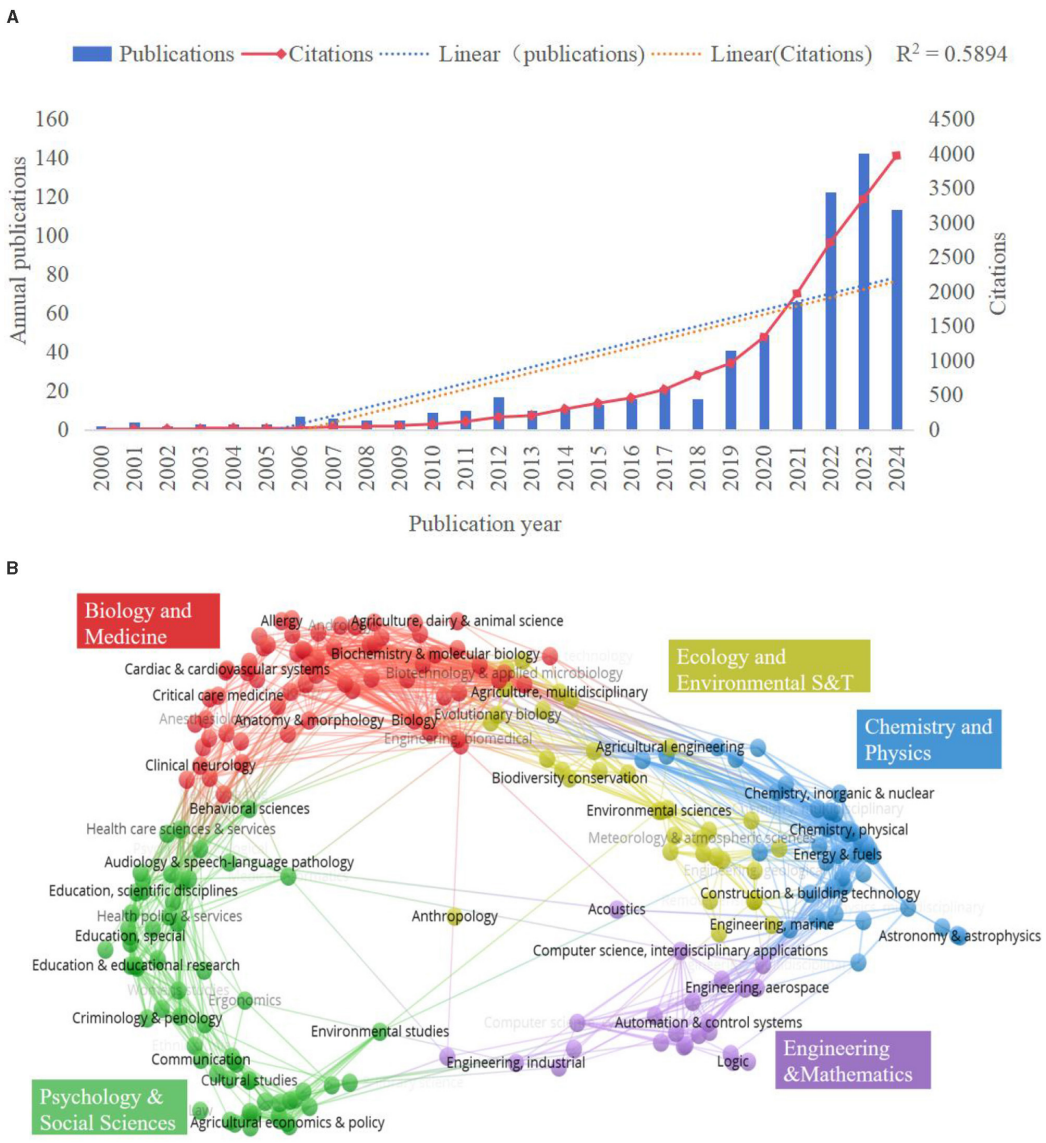
**(A)** Annual publications and citation trends (2000–2024). **(B)** Overlay analysis of the distribution of discipline fields.

The cumulative number of publications and citations over different periods can serve as indicators of the development trends in farmers' green production behavior (16). As illustrated in Figure 3A, the number of publications prior to 2019 remained relatively stable, with none exceeding 30. However, the overall volume of literature exhibited an upward trend. Notably, the number of publications gradually increased between 2019 and 2021. In 2022, there was a significant surge, with publications rising to 122, and peaking at 142 in 2023—an increase of 3.46 times compared to 2019. A linear regression analysis of publication dates and cumulative publications yielded an R^2^ value of 0.5894, indicating a good model fit. This aligns with Price's law of scientific growth, which posits that scientific indicators tend to increase over time (23). By 2024, the frequency of literature citations reached a peak of 3,966, reflecting the widespread recognition and citation of research in this field by the academic community. Therefore, it can be inferred that research related to farmers' green production behavior is experiencing rapid growth, with a continuing increase in publication rates.

Using VOSviewer software, the disciplinary areas related to the literature on farmers' green production behavior were analyzed and superimposed, with the results presented in Figure 3B. In conjunction with Web of Science Categories, the articles on this topic were categorized into 91 distinct disciplinary areas. Table 2 illustrates the number of articles in the top ten disciplines, which include Environmental Sciences (223 articles, 31.99%), Green Sustainable Science and Technology (130 articles, 18.65%), Environmental Studies (116 articles, 16.64%), Agronomy (87 articles, 12.48%), and Agriculture Multidisciplinary (77 articles, 11.05%). The diverse results from these disciplines highlight the significant attention the academic community is giving to the green production behavior of farmers. Furthermore, the use of interdisciplinary and cross-disciplinary research tools not only offers varied perspectives and robust theoretical support for studying farmers' green production behavior but also establishes a foundation for exploring new research avenues.

**Table 2:** Top 10 disciplines in terms of publications.

**Rank**	**WOS categories**	**Record counts**	**%(of 697)**
1	Environmental Sciences	223	31.99
2	Green Sustainable Science Technology	130	18.65
3	Environmental Studies	116	16.64
4	Agronomy	87	12.48
5	Agriculture Multidisciplinary	77	11.05
6	Economics	52	7.46
7	Food Science Technology	48	6.89
8	Engineering Environmental	38	5.45
9	Agricultural Economics Policy	35	5.02
10	Public Environment Occupational Health	29	4.16

#### 3.1.3 Knowledge flow analysis

**Figure 4:**
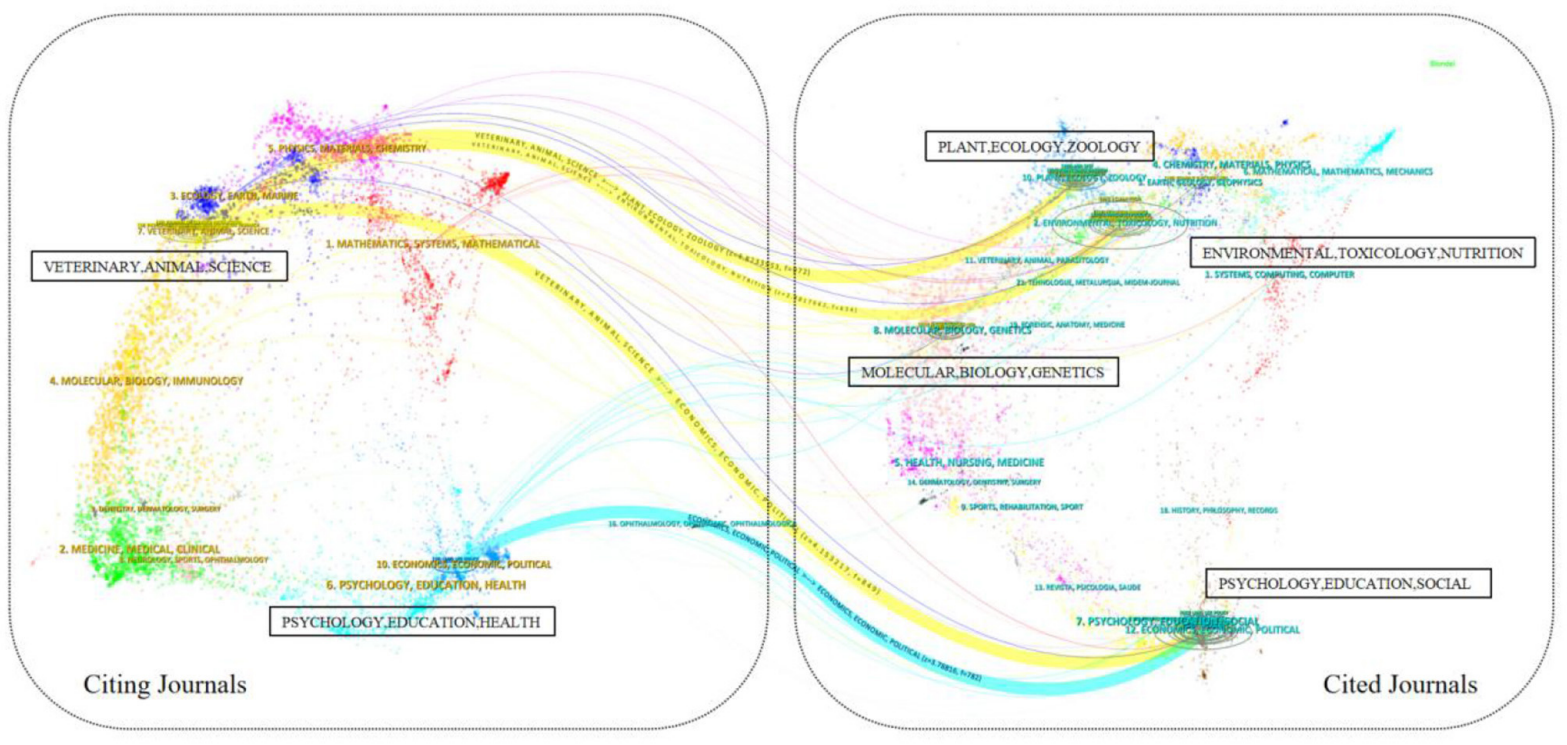
Dual-map overlay analysis mapping.

Dual graph overlay analysis, an advanced feature of CiteSpace, is a new way to display information about the distribution, citation trajectory, and center of gravity drift of papers across disciplines (24). This analysis technique enables researchers to visualize the interactions and connections between journals in different academic fields by displaying two associated network diagrams in the same view. In particular, the left side of the layer is the citing graph, in which each node represents an article that cites other literature, and the connecting lines between the nodes indicate the citation relationship. The right side of the layer is the cited graph, in this view each node represents a piece of literature that is cited by another paper, and the connecting lines between the nodes similarly indicate the citation relationship. The citation graph shows how documents cite other documents, while the cited graph shows how documents are cited by other documents. By comparing the citation graph and the cited graph, researchers can identify key literature, research hotspots, knowledge flow paths, and the distribution of academic influence in the research field. Based on the Z-score algorithm, the literature on green production behavior of farmers was analyzed by overlaying the two maps, as shown in Figure 4.

There are four trajectory lines in Figure 4, which are indicated by the colors of the marked areas, with different colors representing different knowledge groups, and the thickness of the trajectory lines (i.e., citation relations) connecting these areas is directly proportional to the z-score of the citation, i.e., a higher z-score indicates that the citation has been cited more frequently, and thus is represented as a thicker line in the mapping (25). Within the cited areas, the most recorded fields are “ENVIRONMENTAL, TOXICOLOGY, NUTRITION” and “PSYCHOLOGY, EDUCATION, SOCIAL”, implying that these two areas are frequently cited in the study of green production behavior of farmers, and have certain importance and influence. Meanwhile, these two areas have a larger elliptical aspect ratio. A larger ellipse aspect ratio implies that the ellipse is more elongated, highlighting a wider range of research on green production behavior of farmers. In addition, the major citation trajectories originate from these two areas and enter the “PSYCHOLOGY, EDUCATION, HEALTH” and “VETERINARY, ANIMAL, SCIENCE”. The field of “VETERINARY, ANIMAL, SCIENCE” is at the forefront of research. Notably, the citation trajectory from “VETERINARY, ANIMAL, SCIENCE” has a significant z-value (z = 4.82), emphasizing the importance and impact of this development pathway. In the future, “MATHEMATICS, SYSTEMS, MATHEMATICAL” and “MOLECULAR, BIOLOGY, IMMUNOLOGY” may become emerging fields. The field of “ECOLOGY, EARTH, MARINE” may be an emerging field of cutting-edge research.

#### 3.1.4 Analysis of major research journals

Table 3 presents the top 10 journals that have published research on the green production behavior of farmers. Collectively, these journals have published 242 articles, accounting for 34.72% of the total publications. This distribution indicates a lack of a highly concentrated core group of journals in the field, suggesting that publications are relatively dispersed. Notably, the journal SUSTAINABILITY has published 61 papers, significantly more than the second-ranked journal, highlighting its strong focus on the study of green production behavior among farmers. In terms of citation index, the JOURNAL OF CLEANER PRODUCTION, LAND USE POLICY, and AGRICULTURAL SYSTEMS stand out, with citation counts of 1,082, 1,027, and 811, respectively. The average number of citations per article for these journals is 33.81, 57.06, and 57.93, respectively. This data underscores the authority and significance of these three journals in the research on green production behavior of farmers.

**Table 3:** Top 10 authors and cited authors in terms of published papers.

**Rank**	**Journal title**	**Publications**	**Citations**	**Average citations**
1	Sustainability	61	387	6.34
2	Journal of Cleaner Production	32	1,082	33.81
3	Agriculture Basel	31	206	6.65
4	Environmental Science and Pollution Research	20	578	28.9
5	International Journal of Environmental Research and Public Health	20	238	11.9
6	Land Use Policy	18	1,027	57.06
7	Frontiers in Environmental Science	16	154	9.63
8	Frontiers in Sustainable Food Systems	15	96	6.4
9	Land	15	239	15.93
10	Agricultural Systems	14	811	57.93

### 3.2 Research power

#### 3.2.1 Countries and collaborations analysis

**Figure 5:**
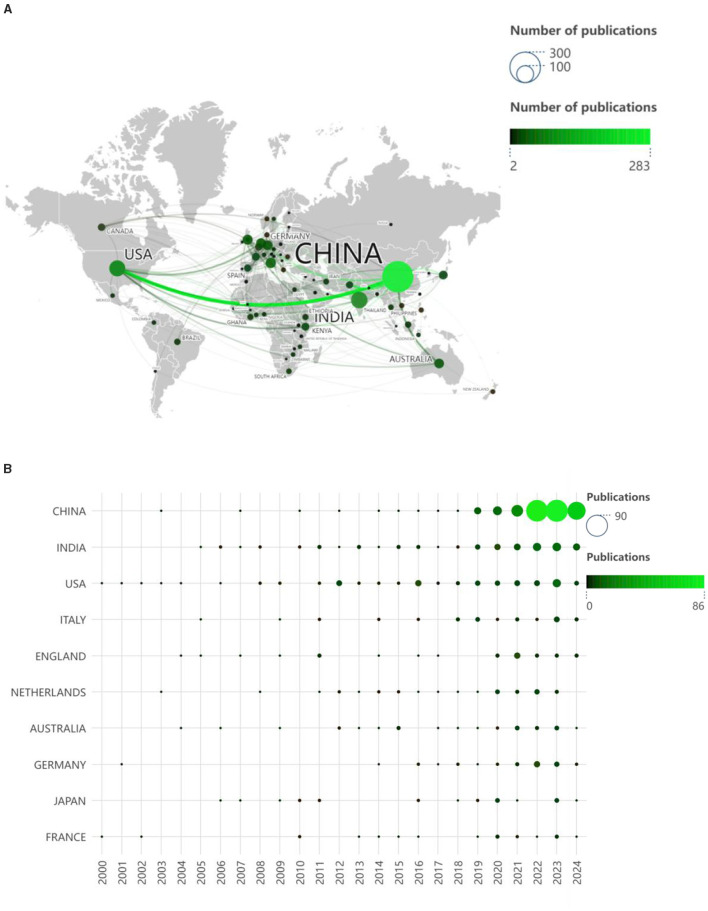
**(A)** National collaboration network. **(B)** Annual volume of publications in the top 10 countries.

Instrumental This analysis method can show the national distribution of research on farmers' green production behavior and the cooperation relationship between countries (26). In Figure 5A, it can be seen that countries' cooperative relationship in the study of farmers' green production behavior. The size of the circle in the graph reflected the number of articles published by each country, and the thickness of the lines between countries indicated the degree of cooperation between countries. The thicker the lines, the closer the cooperation. Generally speaking, the research topic of farmers' green production behavior has received extensive international attention, especially in China and the United States, which not only published a large number of articles, but also had a very close cooperative relationship. Figure 5B shows the top ten countries in terms of annual circulation of studies on green production behavior of farmers. It can be seen that the United States started earlier, and the number is relatively stable. Although China started late, the number has gradually increased incrementally since 2019, and the number of publications far exceeds that of other countries. As can be seen from Table 4, the top 10 countries accounted for 88.81% of the total circulation, with a total of 619 publications. In these papers, developed countries account for the majority, indicating that the research on farmers' green production behavior has become the focus of the global academic community, especially in developed countries, and this issue has received wide attention. At present, China, India and the United States are far more than other countries, respectively 283, 79 and 74, accounting for 40.6%, 11.33% and 10.62%. In addition, these three countries are far more cited than other countries, making outstanding contributions to the field. The Average citations of the United States are the highest, indicating that it has a high influence on the research of farmers' green production behavior. The H-index value of China is the largest, indicating that it is currently at the core of the research in this field.

**Table 4:** The top 10 countries in terms of publications and cooperation.

**Rank**	**Country**	**Publications**	**%(of 697)**	**Citations**	**Average citations**	**H-index**
1	China	283	40.60	4,553	16.09	33
2	India	79	11.33	1,974	24.99	19
3	USA	74	10.62	3,779	51.07	29
4	Italy	31	4.45	691	22.29	15
5	England	29	4.16	1,427	49.21	15
6	Netherlands	29	4.16	1,156	39.86	16
7	Australia	27	3.87	982	36.37	14
8	Germany	27	3.87	553	20.48	12
9	Japan	22	3.16	468	21.27	9
10	France	18	2.58	464	25.78	9

#### 3.2.2 Institutions and authors analysis

The Through in-depth analysis of the number of publications and the number of citations, it can objectively reflect the contribution degree and academic status of the institution or the author in the relevant academic field (27). Tables 5, 6 show the top 10 institutions and authors respectively. In Table 5, higher education institutions play a major role in the research on farmers' green production behavior, among which, among the top institutions of higher learning, NORTHWEST AF UNIVERSITY CHINA (28 articles) and SICHUAN AGRICULTURAL UNIVERSITY (25 articles) led the way. CGIAR has the highest H-index value, which reflects its significant influence on the research topic. It is worth noting that although WAGENINGEN UNIVERSITY RESEARCH is not in the leading position in the number of publications, the average number of citations of its articles is high, reflecting the high level of its research. The results in Table 6 show that Fu XH, Sichuan Agricultural University, and Kumar A, ICAR-ICAR Research Complex for Eastern Region, with Sichuan Agricultural Liu YY from University ranked the top three authors with 9, 8 and 8 articles respectively. In terms of average quotation rate, Nordin SM and Adnan N were significantly higher than other authors, 55.88 and 59.43 respectively, which reflects the high quality and highly recognized research of these two scholars. In addition, Nordin SM has the highest H-index value in the research field of farmers' green production behavior.

**Table 5:** Top 10 institutions in terms of publications.

**Rank**	**Institution**	**Publications**	**Citations**	**Average citations**	**Country**	**H-index**
1	CGIAR	32	1,738	85.56	USA	20
2	Northwest A F University China	28	554	19.79	China	10
3	Indian Council of Agricultural Research ICAR	27	440	16.3	India	10
4	Sichuan Agricultural University	25	316	12.64	China	10
5	Wageningen University Research	18	787	43.72	Netherlands	12
6	China Agricultural University	15	189	12.6	China	6
7	Chinese Academy of Sciences	14	259	18.5	China	10
8	Nanjing Agricultural University	14	386	27.57	China	7
9	Huazhong Agricultural University	13	153	11.77	China	6
10	Beijing Forestry University	11	230	20.91	China	5

**Table 6:** Top 10 authors in terms of publications.

**Rank**	**Author**	**Publications**	**Citations**	**Average citations**	**Country**	**Institution**	**H-index**
1	Fu XH	9	91	10.11	China	Sichuan Agricultural University	6
2	Kumar A	8	115	14.38	India	ICAR—ICAR Research Complex for Eastern Region	5
3	Liu YY	8	78	9.75	China	Sichuan Agricultural University	5
4	Nordin SM	8	447	55.88	Malaysia	Universiti Teknologi Petronas	8
5	Adnan N	7	416	59.43	Saudi Arabia	Prince Mohammad Bin Fahd University	7
6	Li H	7	51	7.29	China	South China Agricultural University	4
7	Liu Y	7	122	17.43	China	Sichuan Agricultural University	5
8	Luo L	7	85	12.14	Finland	University of Helsinki	6
9	Qiao DK	6	77	12.83	China	China Agricultural University	5
10	Kumar R	5	9	1.8	India	ICAR—National Academy of Agricultural Research & Management	2

### 3.3 Knowledge base and theme progress

#### 3.3.1 Research knowledge base

**Figure 6:**
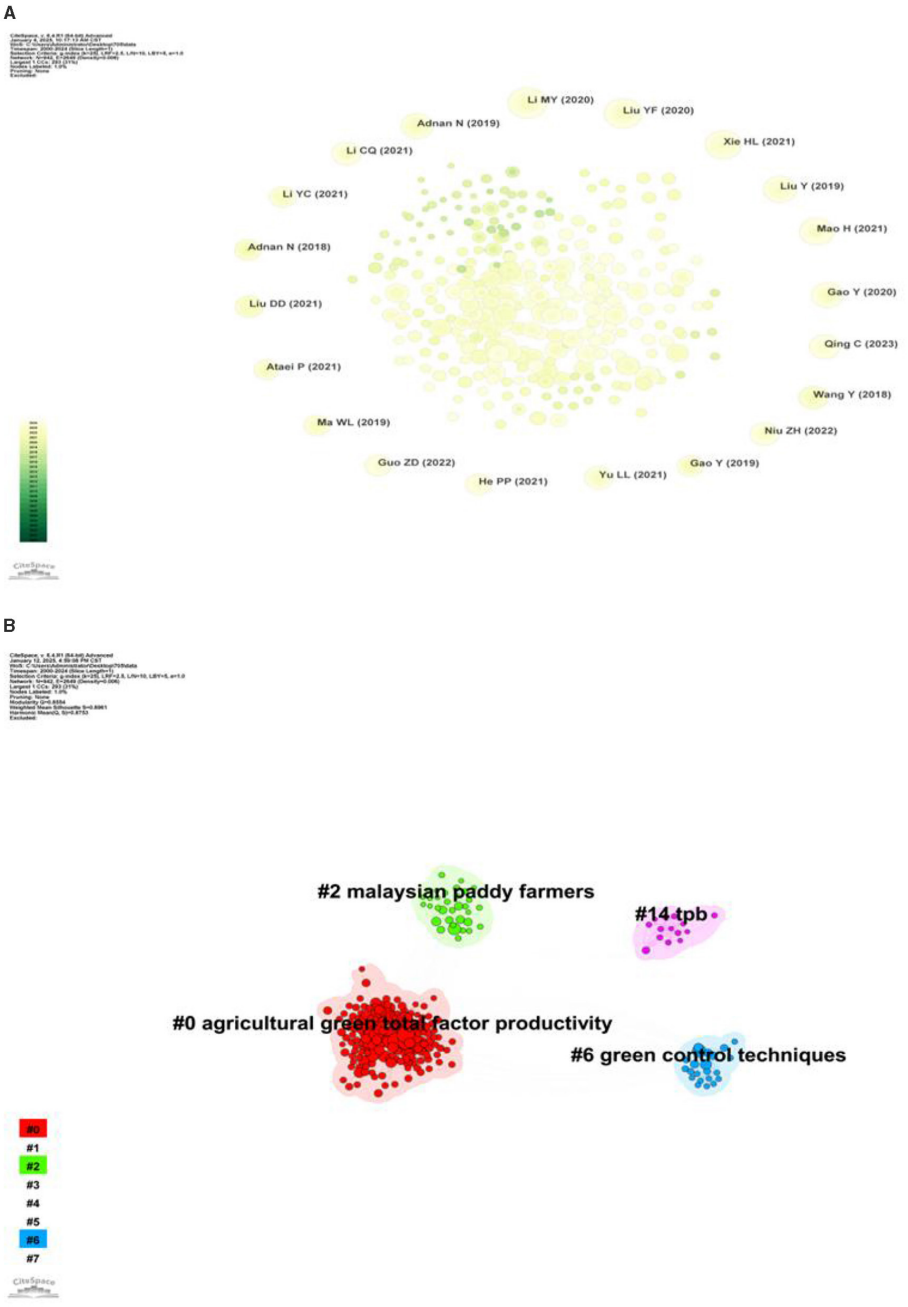
**(A)** Co-citation analysis of references. **(B)** Clustering network analysis of references.

In literature research, if a new literature (cited literature) simultaneously cites two or more old literatures, that is, the cited literature, then a co-citation relationship is formed between these cited literatures (28). Self-reference mapping is an analytical tool that reveals the knowledge structure in a specific domain by studying the reference relationships among literatures (29). This approach focuses specifically on the citation itself, treating each cited article as a node in the network. In this way, a knowledge network can be constructed, which contains the reference relationship of the literature in a specific field, thus revealing the knowledge structure and research progress in the field. Figure 6A shows the co-citation analysis diagram of farmers' green production behavior. The larger the nodes in the diagram, the higher the frequency of co-citation. Cocitation cluster analysis can be used to explore knowledge structure and research boundaries (30). The co-citation clustering analysis of the extant literature on farmers' green production behavior, as depicted in Figure 6B, reveals a clustering quality (Q) value of 0.8554, which exceeds the threshold of 0.3, and an average clustering weight (S) of 0.8961, surpassing the critical value of 0.7. These metrics collectively suggest that the clustering distribution is homogeneously dispersed and exhibits a robust, credible structure. This finding corroborates that the boundaries of the research domain are clearly delineated and that substantial heterogeneity exists within this field. The figure contains four cluster labels, each of which is associated with theme color blocks corresponding to different time slices, among which #0 Agricultural Green Total Factor Productivity is a prominent emerging research topic in this research field.

#### 3.3.2 Seminal literature analysis

This study makes further analysis according to the top 10 nodes in co-citation frequency, as shown in Table 7. According to the research content, it can be divided into three categories. (1) Regarding the driving factors of farmers' adoption of green production behaviors, Li et al. (31) explored the positive effects of farmers' perceived value and perceived income from the perspective of farmers' cognition, while perceived risks negatively affected their implementation of green production behaviors; Xie and Huang (32) used meta-analysis to discuss the relationship between factors such as family characteristics and external environment of farmers and their adoption of environmentally friendly agricultural technologies. By constructing the tobit regression model, Wang et al. (37) empirically analyzed that membership of agricultural cooperatives, organic fertilizer subsidies and farm size played a positive role in farmers' selection of organic fertilizers. Liu et al. (68) explored the impact of technical training on the adoption of low-carbon management practices by rice farmers; Mao et al. (33) believed that time preference significantly reduced the adoption rate of green technology among farmers, and found that farmers with larger production and operation scale were more likely to adopt green production technology. Gao et al. used the scoring matching method to explore that the new agricultural technology extension mode can improve the level of farmers' technology adoption, and has different benefits for different groups of farmers (34).

**Table 7:** Nodal information of top 10 cited literature.

**Rank**	**References**	**Co-citation frequency**	**Article title**	**Cluster ID**
1	Li et al. (31)	37	Factors affecting the willingness of agricultural green production from the perspective of farmers' perceptions	0
2	Liu et al. (67)	34	An evaluation of China's agricultural green production: 1978–2017	0
3	Xie and Huang (32)	29	Influencing factors of farmers' adoption of pro-environmental agricultural technologies in China: Meta-analysis	0
4	Adnan et al. (38)	28	A state-of-the-art review on facilitating sustainable agriculture through green fertilizer technology adoption: Assessing farmers behavior	0
5	Mao et al. (33)	28	Time Preferences and green agricultural technology adoption: Field evidence from rice farmers in China	0
6	Liu et al. (68)	28	Technical training and rice farmers' adoption of low-carbon management practices: the case of soil testing and formulated fertilization technologies in Hubei, China	0
7	Gao et al. (34)	23	Influence of a new agricultural technology extension mode on farmers' technology adoption behavior in China	0
8	Qing et al. (35)	22	Impact of outsourced machinery services on farmers' green production behavior: evidence from Chinese rice farmers	0
9	Niu et al. (36)	20	Peer effects, attention allocation and farmers' adoption of cleaner production technology: taking green control techniques as an example	0
10	Wang et al. (37)	20	What could promote farmers to replace chemical fertilizers with organic fertilizers?	0

Qing et al. (35) made an empirical analysis that mechanical outsourcing services could significantly influence 1,080 rice farmers in Sichuan Province, China to adopt green production behaviors. In addition, Niu et al. (36) adopted IV-Probit model and found that the adoption of green control technology by farmers would be affected by peer effect. (2) In terms of the development of theoretical models for farmers' green production behaviors, Adnan et al. (38) combined with theoretical frameworks such as Diffusion of innovation (DOI), Theory of Planned Behavior (TPB) and Technology acceptance model (TAM) to write a literature review to clarify the key factors of green fertilizer technology (GFT) adoption by rice farmers in Malaysia. (3) Regarding the implementation and evaluation of green production behaviors, Liu et al. (67) empirically determined the gap between the current situation and the target value of China's agricultural green production, as well as the vertical and spatial evolution of China's agricultural green production level from five dimensions of supply capacity, resource utilization, environmental quality, ecosystem maintenance and farmers' livelihood.

#### 3.3.3 Research thematic progress

**Figure 7:**
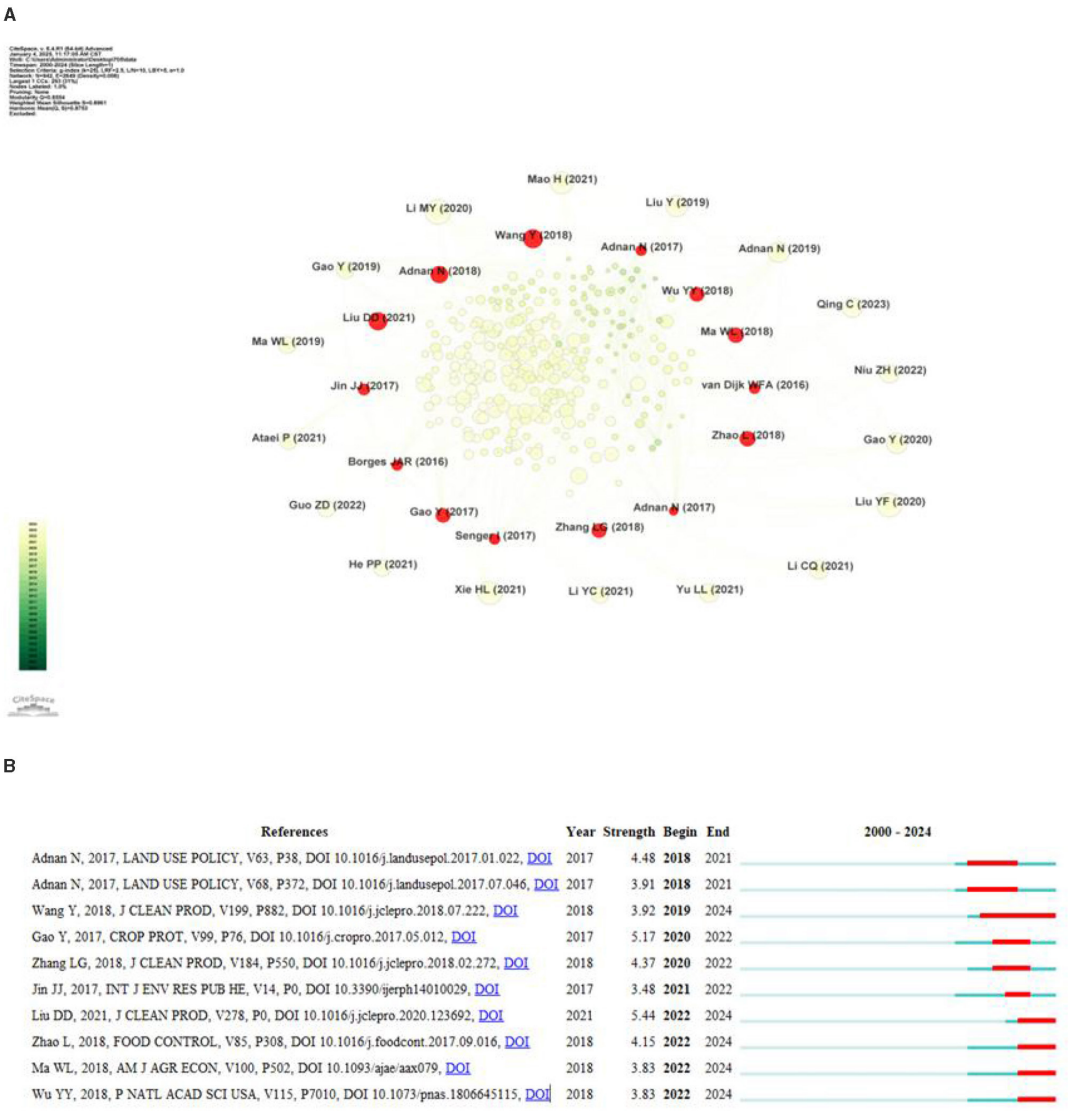
**(A)** Burst detection of co-citation. **(B)** The top 10 references with the strongest citation bursts.

Burst detection refers to a phenomenon in which the citation frequency of certain documents or terms suddenly increases within a specific period of time. By analyzing the literature that is suddenly cited, we can identify the current research hotspots and trends of the discipline, and help researchers grasp the development direction of the discipline (39). Figure 7 shows the burst citation mapping of the research on farmers' green production behavior, where the burst citations are represented by red nodes in Figures 7A, B shows the top 10 literature on burst intensity. In this study, we will conduct an in-depth analysis of the literature with high outbreak intensity in combination with the literature review method, so as to reveal its contribution to the study of farmers' green production behavior and its position in the evolution of knowledge.

As can be seen from Figure 7, the previous research focused on theoretical analysis and explored the applicability of various theoretical frameworks to explain farmers' green production behavior. During this period, a high-intensity literature appeared: Adnan et al. (66) conducted theoretical and applied research on farmers' green production behavior, and based on the conceptual framework of planned behavior theory (TPB), rational behavior theory (TRA) and expected utility theory (EUT), explored the driving factors for Malaysian rice farmers to adopt green production behavior. At the same time, Adnan et al. (65) also combined the Theory of Planning Behavior (TPB) and the Technology Acceptance Model (TAM) to explore the impact of psychological factors such as farmers' perceived ease of use, perceived usefulness, and subjective norms on their adoption of sustainable agricultural practices. Notably, Gao et al. (40) (Strength = 5.17) also found that perceived ease of use and usefulness of technology can also have a positive impact on the adoption of green control technology behavior on family farms. These studies provide an important theoretical basis for understanding the behavior patterns of farmers in green production.

Then, the research theme began to try to combine a variety of empirical models to clarify the factors influencing farmers' adoption of green production behaviors. Based on the prospect theory and field theory, Wang et al. (37) built a tobit regression model and found that the membership of agricultural cooperatives, fertilizer subsidies and business scale had a positive effect on farmers' choice of organic fertilizer. Zhang et al. (41) found that farmers' awareness of the environment, pesticide residues and the quality of agricultural products had a positive impact on their willingness to adopt environmentally friendly agricultural production, rather than a negative impact on agricultural income. Zhao et al. (42) found that market incentives can significantly affect the pesticide application behavior of vegetable farmers.

Recent studies mainly focus on the benefits brought by farmers' green production. During this period, there was also a literature with high explosive intensity: Liu et al. (43) (Strength = 5.44) evaluated China's agricultural green total factor productivity based on carbon emissions, and believed that agricultural green production could promote China's high-quality economic development.

To sum up, the current research has evolved from focusing on the applicability of different theoretical frameworks to explain farmers' green production behaviors in the early stage, to analyzing the factors influencing farmers' adoption of green production behaviors with various empirical models, and to evaluating the benefits brought by farmers' green production. These studies provide important theoretical basis and empirical support for in-depth understanding of farmers' green production behaviors.

### 3.4 Research hotspots, evolutionary trends, and mass distribution

#### 3.4.1 Core keyword analysis

Core keywords are a brief expression of the core content of an article, a precise distillation and summary of the main idea of the literature (44). In CiteSpace, centrality is an important metric to measure the importance of nodes in the network. Specifically, nodes with a centrality value greater than 0.1 are considered key nodes, a criterion that helps identify nodes with high influence in the academic network. The analysis of high-frequency keywords and the examination of centrality metrics can effectively reveal hot topics within the research field (45). In this study, according to the key eat analysis in CiteSpace, the keywords are ranked according to their frequency of occurrence as well as centrality, in which the top 10 keywords have been listed in Table 8 in descending order. The results in Table 8 show that the keyword with the highest frequency is “adoption” (89), which indicates that in the study of green production behavior of farmers, scholars are generally concerned about the process of adoption of green production technology, influencing factors and their effects. This may include farmers' acceptance of green production technologies, motivations for adoption, barriers, and strategies to promote adoption. The second is “green revolution” (68) which suggests that researchers are exploring farmers' green production behaviors in the broader context of agricultural development and transformation. The green revolution involves not only technological innovations, but also changes in agricultural production methods and the far-reaching environmental and social impacts of these changes (46). In addition, the keyword “attitudes” has a Centrality of 0.1, suggesting that the adoption of green production behaviors by farmers is the focus of current research.

**Table 8:** Top 10 keywords by frequency and centrality.

**Rank**	**Keyword**	**Frequency**	**Rank**	**Keyword**	**Centrality**
1	Adoption	89	1	Management	0.19
2	Green revolution	71	2	Green revolution	0.14
3	Impact	68	3	Technology	0.14
4	Technology	67	4	Farmers	0.13
5	Management	65	5	Attitudes	0.11
6	Agriculture	64	6	Yield	0.08
7	Farmers	63	7	Drip irrigation	0.08
8	Behavior	62	8	Technology adoption	0.07
9	Technology adoption	50	9	Climate change	0.07
10	Food security	40	10	Beliefs	0.07

#### 3.4.2 Analysis of research hotspots

**Figure 8:**
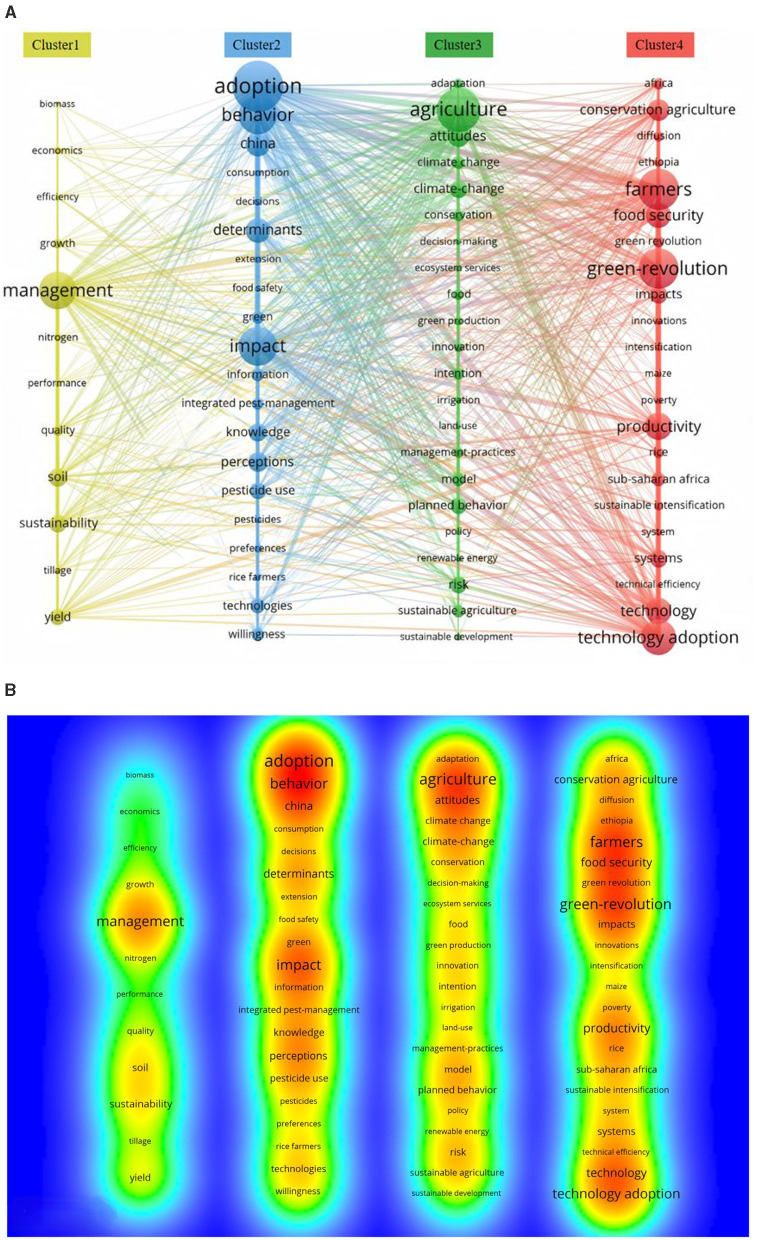
**(A)** Co-occurrence clustering network. **(B)** Keyword density.

When VOSviewer performs keyword co-occurrence analysis, the software organizes keywords into groups or clusters based on their intrinsic connections and frequency of occurrence, thus highlighting hot topics in the research field. The strength of connections between keywords reveals the interconnectedness of different topics. In order to improve the accuracy of the analysis, only keywords labeled by the authors in the literature were considered in this study. This approach helped to provide an in-depth understanding of the structure of the research field and to identify the core research themes. The keyword frequency of VOSviewer software was set to 10, which resulted in a keyword clustering map for the study of green production behavior of farmers (Figure 8). By comprehensively analyzing the keyword co-occurrence clustering network (Figure 8A) and the corresponding density distribution map (Figure 8B), we can explore the following four groups of clustering themes in depth.

Cluster #1—The study focuses on the impact of adoption of green production practices by farmers. Key words such as sustainability, economics, soil, yield, quality, and efficiency suggest that the adoption of green production behaviors by farmers not only contributes to higher economic returns from agricultural production, but also improves crop quantity and quality, increases production efficiency, and promotes soil health and environmental sustainability. Together, these factors provide a solid foundation for the transformation of agricultural production.

Cluster #2—The study of the adoption of green production behaviors of farmers and the factors influencing them is a hot topic, which involves how farmers make decisions to adopt green production technologies and the factors influencing these decisions, such as access to information, level of knowledge, and perceptions. In addition, the keyword “China” indicates that China is a country that pays more attention to the green production behavior of farmers. Meanwhile, “consumption” and “food safety” point to the impact of green production on consumption and food safety. Therefore, an in-depth discussion on how farmers accept, adopt and effectively implement green production behaviors is crucial for maintaining the agricultural production environment. This will not only help to increase food production, but also promote sustainable agricultural development.

Cluster #3—This cluster focuses on Farmers' Green Production Practices, which focuses on how agriculture can be practiced in the context of environmental change, especially climate change, through green and sustainable agricultural practices, including irrigation, land-use, ecosystem services, sustainable agriculture, and management-practices, among others. These practices are essential for improving the sustainability of agricultural production.

Cluster #4—This cluster may explore the impact of the Green Revolution on technology adoption in agriculture and how these technologies can increase productivity and innovation, with key keywords such as Green Revolution and Technology Adoption appearing, suggesting that it emphasizes the role of technological innovation and technology adoption in increasing agricultural productivity and sustainability, and how sustainable intensification can be achieved through a systematic approach.

#### 3.4.3 Evolutionary trend analysis

**Figure 9:**
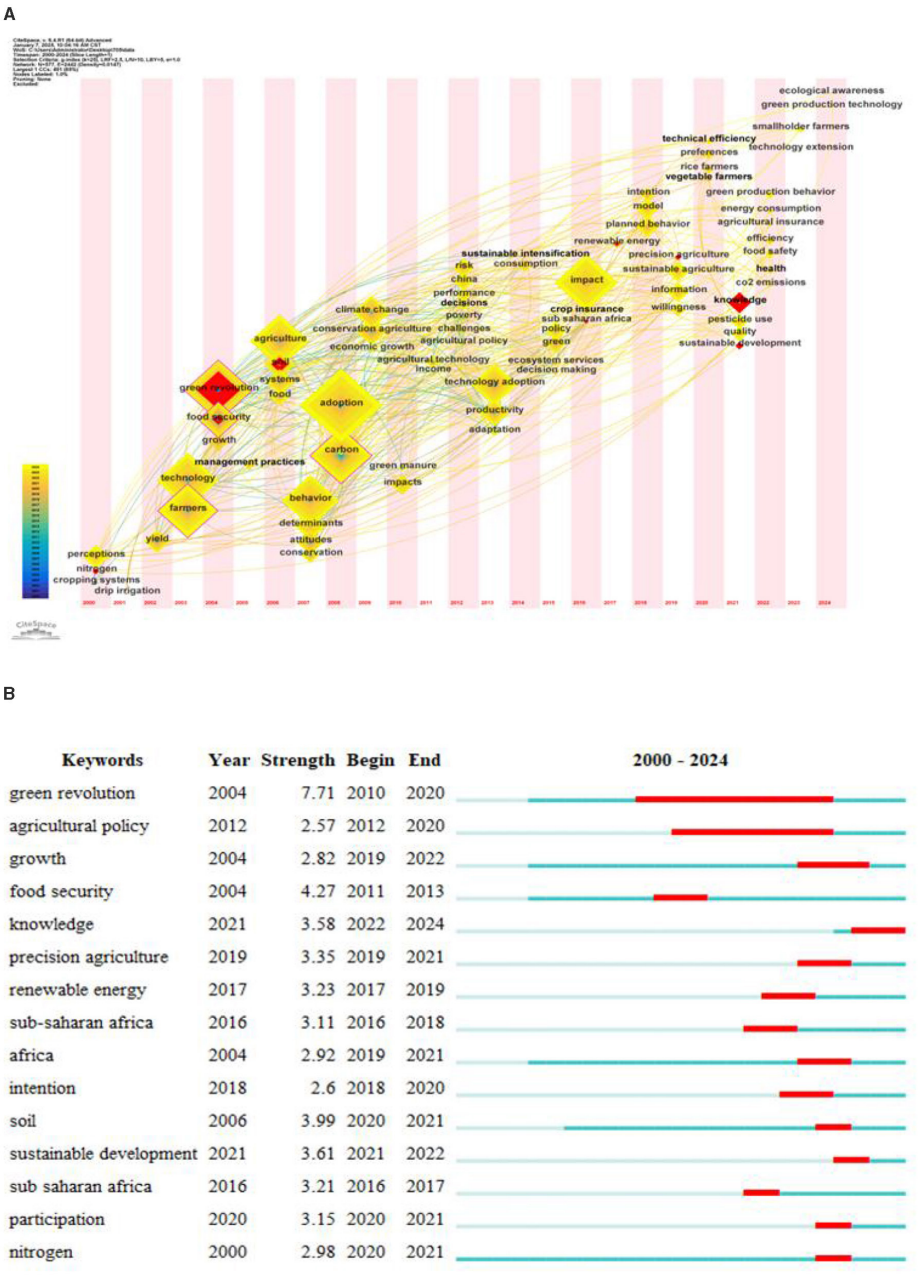
**(A)** Reflecting the frequency and time of first appearance of keywords in the study. **(B)** Top 15 keywords with the strongest citation bursts.

In order to explore in depth the evolutionary trend of the research field of green production behavior of farmers, we conducted a statistical analysis of the emergence time of the keywords. By using CiteSpace software, we constructed time-zone evolutionary mappings for the keywords separately (Figure 9A) and identified the emergent keywords in this research field (Figure 9B). The time zone mapping can identify the research hotspots in a specific time period by analyzing the keywords that appear frequently in that period, and can provide an understanding of the evolutionary path of the research area and the possible future development direction by observing the changes of the keywords over time (69). Figure 8B shows the top 10 keywords in terms of burst intensity, the red markers refer to the high-frequency citation terms and their concentrated burst intensity in a specific year, and these concentrated burst keywords reveal the core focus and evolutionary trend of research topics in different periods (47). By integrating the information of time zone mapping and keyword outbreaks, a more objective and precise research analysis can be provided (48).

Combined with Figures 9A, B, early research on green production behavior of farmers mainly focused on factors such as “green revolution”, “agriculture”, “food security” and “soil”. “food security” and “soil”. This shows that early studies have paid attention to the impact of the green revolution on agriculture (49), food security, and the environment (50). In addition, “nitrogen”, “yield”, and “cropping systems” are also high-frequency words at this stage, reflecting the concern about the use of nitrogen fertilizer and the optimization of cropping systems. and cropping system optimization concerns. This may be closely related to efforts to improve crop yields and sustainable agricultural practices. Subsequently, research began to focus on factors influencing farmers' adoption of green production behaviors and attitudes, such as RISK (51), INCOME, POLICY (52), and CROP INSURANCE (53). It is noteworthy that “climate change” appeared in 2009, indicating that green production by farmers is closely related to global climate change. In addition, the appearance of keywords such as “China”, “Africa”, and “Sub-Saharan Africa” shows that the literature in this period explores the differences in the adoption of green production technologies by farmers in different regions and farm households. With economic development and rising living standards, recent research has shifted to the areas of “efficiency”, “health”, “food safety” and “sustainable development”, focusing on the benefits behind the implementation of green production practices by farmers. “health”, “food safety” and “sustainable development”, focusing on the benefits behind the implementation of green production behaviors by farmers. Translated with DeepL.com (free version). In addition, the emergence of keywords such as “impact” and “knowledge” reflects the assessment of the environmental and social impacts of farmers' green production behaviors and how knowledge dissemination can contribute to sustainable agricultural practices.

In summary, the trend of research on green production behavior of farmers has evolved from early agricultural production technologies and methods to the initial exploration of farmers' adoption behavior, attitudes, and influencing factors on green production. In recent years, research has begun to gradually focus on the decision-making process of farmers and the effects of implementing green production behaviors. This study not only reflects the diversity and complexity of the field, but also demonstrates a comprehensive and in-depth understanding of farmers' green production behaviors.

#### 3.4.4 Research quality distribution

To elucidate the distribution of research quality within the domain of farmers' green production behavior, we employed the strategic coordinate diagram analysis of research themes. This method enabled us to quantify and depict the internal development trajectories and interrelationships among diverse research themes. The Strategic Diagram (SD) was proposed by Law et al. in 1988 to describe the extent to which themes within a research area are interconnected and interact with each other (54). In the two-dimensional strategic coordinates, Centrality is the horizontal axis, which is used to measure the closeness of the interconnection between each category's subject matter and other categories' subject matter, and the larger the value, the more central this subject matter tends to be in the whole research work; Density, the vertical axis, is used to measure the closeness of the subject terms within each category, and it indicates the ability of the category to sustain itself and develop itself. By assigning values to each research hotspot and pointing to a certain quadrant, the strategic coordinates can summarize the development of different topics within a field (55).

**Figure 10:**
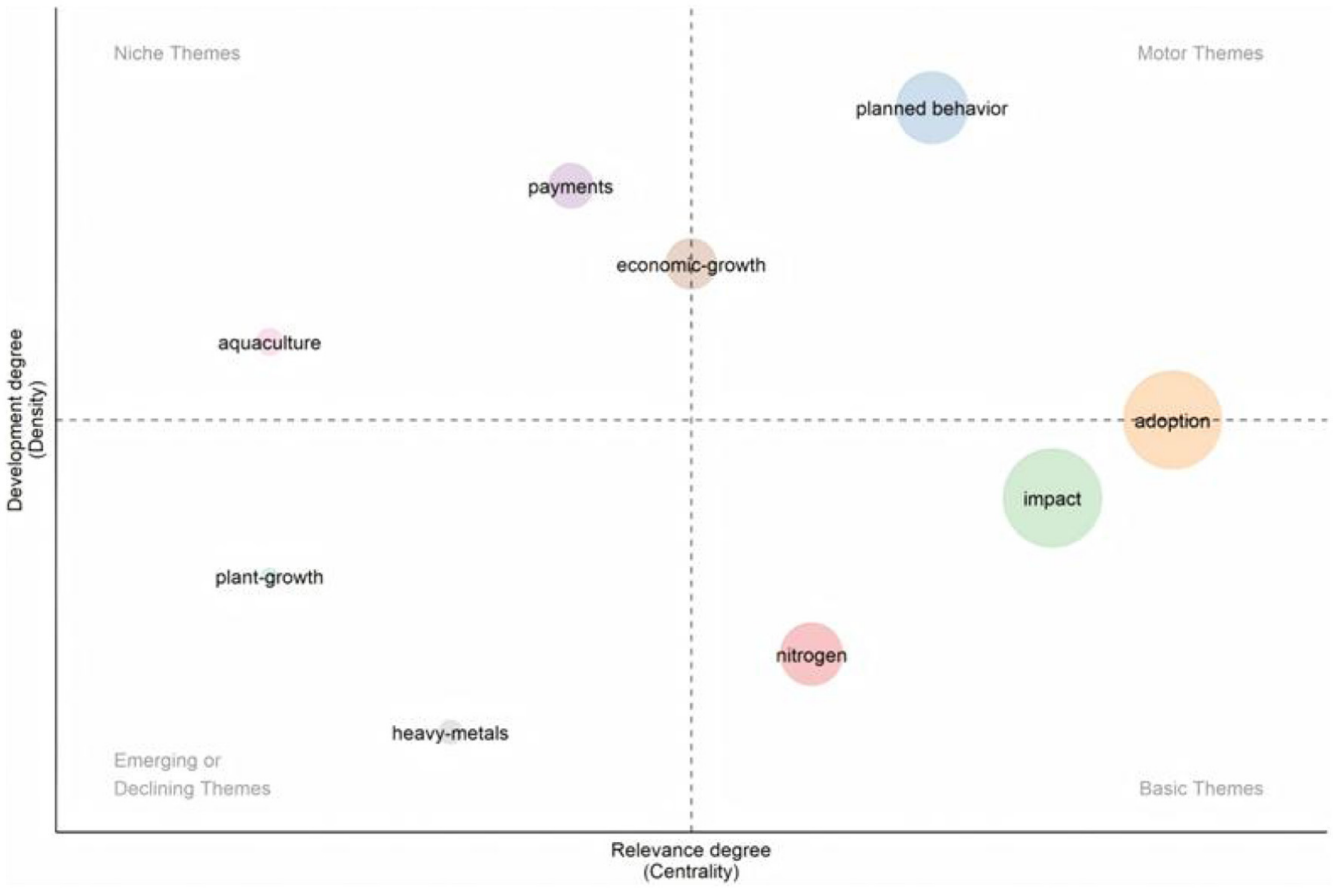
Strategic diagram of theme clusters.

In the present study, a total of 57 core keywords (with a frequency of ≥5) were systematically categorized into nine thematic clusters via cluster analysis, which was subsequently validated through manual verification (Table 9). Additionally, the centripetal degree (represented on the X-axis) and density (represented on the Y-axis) of each keyword were accurately computed (Table 10). Finally, the class clusters composed of high-frequency keywords were displayed in the strategy map, and the strategic coordinate map of the research hotspots of green production behavior of farmers from 2000 to 2024 was drawn (shown in Figure 10), and the structure and development trend of the current research on green production behavior of farmers were analyzed as a result.

**Table 9:** Classification of high frequency keywords.

**Cluster ID**	**Keywords**
1	Planned behavior, intention, decision-making, renewable energy biological-control, fertilizer technology, user acceptance, conservation practices, information-technology, dairy farmers
2	Adoption, behavior, agriculture, determinants, attitudes, conservation agriculture, knowledge, perceptions, risk, technologies
3	Impact, management, green-revolution, farmers, technology, soil, technology adoption, productivity, systems
4	Nitrogen, biomass, waste, cropping, systems, energy, yields, crops, waste, hunger, manure
5	Economic-growth, energy-consumption, CO_2_ emissions, agricultural productivity, carbon emissions, evolution, farm size
6	Payments, choices, agricultural policy, cost, crop insurance, programs
7	Aquaculture, methane
8	Plant-growth
9	Heavy-metals

**Table 10:** Strategic coordinates of thematic clusters.

**Cluster ID**	**Keyword counts**	**Rank centrality**	**Ran density**
1	10	7	9
2	10	9	5
3	10	8	4
4	10	6	2
5	7	5	7
6	6	4	8
7	2	1.5	6
8	1	1.5	3
9	1	3	1

As shown in Figure 10, (1) the theme clustering of “planned behavior” is located in the first quadrant, showing high centrality and high density. This indicates that these themes not only have strong internal cohesion, but also are closely connected with other themes, showing that they have a high degree of maturity in development, have formed systematic research contents or directions, and have had a significant impact on other themes. These themes occupy a central position in the research field of farmers' green production behavior and have a broad development prospect. (2) The thematic domains of “payments” and “aquaculture” are situated within the second quadrant, characterized by elevated density yet diminished centrality. This indicates that these themes have strong internal connections but weak connections with external themes, which suggests that although they have received extensive attention from researchers, they exist more as an independent system and show a certain degree of independence. Consequently, it is imperative for future research endeavors to delve into the intricate synergies and cross-disciplinary applications of these themes in conjunction with other relevant thematic areas. (3) The clusters of “plant-growth” and “heavy-metals” are located in the third quadrant, with low centrality and density. These themes are loosely connected internally and weakly connected to other themes, indicating that their development is still immature and they belong to marginal and niche themes, which need to be further researched. (4) The theme clusters of “impact”, “adoption” and “nitrogen” are located in the fourth quadrant, with higher centrality but lower density. Although these themes are closely linked to other research themes, their internal cohesion is weak, indicating that these themes are not yet fully developed on their own and their cores are immature. Nevertheless, they have an important place in the field of research on farmers' green production behavior and have potential for future exploration. (5) The theme clustering of “Economic-Growth” is located near the center, suggesting that it may be a moderately developed and relevant theme. This may mean that the relationship between economic growth and green production behavior is an important area of research, but more research may be needed to determine its specific role.

## 4 Discussion

Green production behavior is gradually becoming a research hotspot in the field of agriculture, and the adoption of green production behavior by farmers not only helps to reduce the waste of resources and damage to the environment, but also plays a crucial role in improving the ecological environment and maintaining the ecological balance. In order to explore the research progress in this field, we conducted a comprehensive bibliometric analysis of the literature related to green production behaviors of farmers with the help of Web of Science (WOS) Core Collection database. By using visualization tools such as CiteSpace and VOSviewer, we have thoroughly combed and analyzed the number of publications, major contributing countries, research institutions, published journals and authors of these studies. In addition, we delved into the distribution of keywords, cited journals, and references to identify research hotspots and their evolutionary trends over different time periods.

Of the 604 publications retrieved from the WoSCC database related to green production behavior of farmers, the number of publications increased from 2 in 2000 to 144 in 2023.The year 2019 serves as a pivotal demarcation point, distinguishing between the phases of slow and rapid growth. This suggests that the volume of scholarly publications focused on the green production behavior of farmers has been progressively increasing, garnering significant attention and popularity. The 255 journals publishing on green production behavior of farmers cover 88 different field disciplines such as Environmental Sciences, Green Sustainable Science Technology, and Environmental Studies. The nine most prolific journals collectively constituted 26.32% of the total publications, amounting to 159 articles. This underscores their significant role in shaping the discourse and contributing substantially to the advancement of research on the green production behavior of farmers. Compared to other journals, Sustainability has a particularly high number of publications (44, or 7.3%) and a 2023 impact factor of 3.3. This is attributed to the fact that the Sustainability journal focuses on research on human environmental, cultural, economic, and social sustainability, and that its theme is highly compatible with research on green production behaviors of farmers, which makes it ideal for research in this area. It is an ideal platform for research in this field. In addition, as an open access journal, the free access mode of Sustainability effectively expands the dissemination and influence of the articles. At the same time, its efficient review process allows for quick feedback and publication, which meets the needs of researchers and attracts a large number of scholars to submit their papers for publication. It is worth noting that JOURNAL OF CLEANER PRODUCTION does not have the same number of publications as Sustainability, but it has the highest number of citations (990) among these nine journals, which demonstrates that JOURNAL OF CLEANER PRODUCTION is in the field of research on the green production behavior of farm households' Influence. Additionally, the nine most prolific journals in terms of publication output were all based in Europe. This underscores the fact that Europe has established a robust research platform that has significantly contributed to the advancement of studies on farmers' green production behavior.

Among the 90 countries examined, China, India, and the United States exhibited the most substantial publication output, thereby emerging as the predominant forces propelling research advancements in the domain of sustainable agricultural. It is worth noting that the trend in the number of national publications shows that China has a remote number of publications from other countries and has made greater progress in this research area in recent years, indicating its growing research capacity and the high priority it places on the agri-environment. This is mainly due to the fact that China has been pursuing a policy of chemical fertilizer and pesticide reduction from the beginning of 2015 to the present day, which has led to a great deal of attention being paid to green production in agriculture (56). In addition, although the United States has a slightly lower number of publications than India, the United States collaborates more with other countries than India, and in particular, the United States and China collaborate more closely. Among the top 10 institutions in terms of the number of publications, China has the most institutions (7), and most of them are Chinese higher education institutions, which indicates that Chinese higher education institutions are active in the field of research on green production behaviors of farmers and have made great contributions. Interestingly, among the top 10 authors in terms of the number of publications, 6 are also scholars from Chinese institutions of higher education, again reflecting that Chinese scholars have paid high attention to this field.

This research elucidates the epistemological framework and thematic evolution within the domain of farmers' green production behaviors by conducting a comprehensive review of seminal literature. The application of self-referential network and cluster methodologies delineates the structural thematic contours of the study, elucidating the distinctions and demarcations within the research domain. Moreover, burst detection analysis offers a salient vantage point for discerning the dynamic progression of thematic trends within the domain of green production behavior among farmers. The development and innovation of theoretical models underpinned this study. The researchers addressed theoretical limitations by deepening and expanding existing theories, thereby enhancing the explanatory power of the constructed models. For example, Xianyu et al. (57) constructed an analytical framework of farmers' cognition-social norms and personal norms-green production behavior based on the theory of planned behavior, norm activation theory, and government regulation theory to explore whether farmers' cognition influences their green production behavior. Xu et al. (58) extended the theory of planned behavior by combining the theory of normative planning to constructed the NAM-TPB model, which greatly improved the prediction of farmers' green production behavior. In the wake of technological progress and the integration of novel technological advancements, a plethora of nascent research agendas have surfaced. Within this context, the scholarly community has demonstrated a growing preoccupation with the extent to which farmers embrace and utilize these emerging technologies. For instance, the research conducted by Dai and Cheng (59) underscored that the key determinants of farmers' adoption of green production practices were their perceptions of the technology's utility and ease of application. This discovery has provided a significant theoretical underpinning for subsequent scholarly inquiries. In addition, the diversification of research methods and innovations in evaluation techniques, such as the use of SEM (11), logistic (60), probit-ISM (61), etc., have enhanced the rigor and reliability of the research results, allowing for a more precise identification of the factors and mechanisms influencing the adoption of green production behaviors by farmers. In summary, the co-citation analysis of the scholarly literature concerning the green production behaviors of farm households elucidates the increasing sophistication of theoretical frameworks and empirical investigations into emerging technologies. This analytical approach underscores the critical role of methodological advancements and evaluative innovations in unraveling the intricacies of complex socio-economic phenomena. These findings are critical in guiding farmers to embrace new agricultural technologies.

Keywords are indicative of publications, and to a certain extent they can reflect the research hotspots in a specific field (62). We conducted a comprehensive analysis of keyword co-occurrence, clustering, and outburst, and found important keywords such as adoption, green revolution, attitudes and other important keywords. The co-occurrence clustering analysis of keywords in the relevant literature has identified several prominent research hotspots within the domain of green production behaviors among farm households. These hotspots encompass the multifaceted impact of farmers' adoption of green production behaviors, the identification and analysis of influencing factors, the exploration of diverse green production methods, and the influence of the green revolution on the uptake of new agricultural technologies. Time-zone evolution mapping and emergent keyword analysis further revealed the evolutionary trend of research hotspots. Early studies focused on the impacts of the Green Revolution on agriculture, food security, and the environment, which demonstrated the beginning of the academic community's attention to the fact that changes in agricultural production methods can affect the environment. Subsequently, the research was extended to the attitude of farmers to adopt green production behavior and the influencing factors, which shows that the research perspective has shifted from the technical level to the behavioral level, and started to pay attention to the subjective willingness of farmers and the external influencing factors, such as the individual characteristics (58), capital endowment (10), the market environment (11), and government policies (63). In recent years, research has increasingly focused on the implementation effects of green production behaviors, which further shows the depth of research, not only focusing on why farmers adopt green production behaviors, but also beginning to assess the actual effects of these behaviors, such as the impact on economic growth, increased social returns, and ecological improvement (64). This shift reflects the increased emphasis on sustainable development and environmental protection, as well as the increased demand for assessments of the effects of green transformation in agriculture. Overall, this process reflects the evolution of research from technology to behavior to impact assessment, and shows the increasing comprehensiveness and depth of research on green production behavior in agriculture. Moreover, the strategically coordinated mapping analysis provides a lucid illustration of the evolution and interrelationships among various research themes. The high centrality and density of the theme “planned behavior” indicate the advanced stage of research maturity in this area and its substantial impact on other thematic domains. The themes “payments” and “aquaculture” show independent research trends. The themes “plant-growth” and “heavy-metals” are not yet mature, but they have potential for development. The themes “impact”, “adoption” and “nitrogen” are closely related to the other themes and show a core of immaturity but great potential.

In summary, the research trend of green production behavior of farm households is diversified and dynamic, reflecting the in-depth academic discussions on sustainable agricultural development in different socio-economic contexts. Driven by modern agricultural technologies and environmental policies, research should pay sustained attention to the application of green production practices in agriculture and explore the mechanisms of how farmers adopt and effectively implement these practices. This not only helps to improve the eco-efficiency and economic efficiency of agricultural production, but also promotes the sustainable development of agriculture.

In order to further promote the research and practice of green production behavior of farmers and promote the sustainable development of agriculture, future research can be carried out in the following aspects:

(1) Interdisciplinary research and international cooperation. The study of green production behavior of farmers involves multidisciplinary fields, which is complex and interdisciplinary. In the future, interdisciplinary research teams should be constructed to integrate multidisciplinary theories and methods, break down disciplinary barriers, promote knowledge integration and innovation, and provide comprehensive solutions for sustainable agricultural development. Meanwhile, given the different strengths of different countries and regions in green production practices and research, international cooperation and experience exchange are crucial. Global knowledge sharing and technology transfer can be promoted through international cooperation projects, academic conferences and personnel exchanges to facilitate the promotion and application of green production practices. In addition, research should be closely integrated with policy formulation and practical needs, and cooperate in depth with government departments, agricultural enterprises and other stakeholders, so as to transform research results into policy measures and practical actions, and help realize the goal of sustainable development of global agriculture.
(2) Deepening and Refinement of Theoretical Models. Although existing theories such as the Theory of Planned Behavior (TPB) and the Technology Acceptance Model (TAM) provide the basis for the study of farmers' green production behavior, they are still insufficient in explaining its complexity and dynamics. Future research needs to be deepened in two ways: first, integrating multidisciplinary theories such as psychology, sociology, economics and environmental sciences to build a more comprehensive theoretical framework to accurately analyze the driving factors and influencing mechanisms of farmers' green production behavior; second, breaking through the limitations of short-term research and analyzing the evolution and sustainability of farmers' long-term behavior through longitudinal studies and dynamic models, in order to provide a more prospective and targeted support to the policy formulation. The second is to break through the limitations of short-term research and analyze the long-term evolution and persistence of farmers' behavior through longitudinal research and dynamic models, so as to provide more prospective and targeted support for policy formulation.
(3) Improving farmers' acceptance and application effectiveness of new agricultural technologies. Despite the development of agricultural technology and the increasing importance of emerging technologies such as precision agriculture technology and smart devices for green production, the acceptance and application effect of new technologies by farmers varies widely, which affects the effectiveness of technology promotion. Future research should focus on farmers' user experience and acceptance of new technologies, analyze the barriers they face in adopting new technologies, such as technological complexity, cost inputs, and risk perceptions, and explore strategies to promote technology adoption, such as technical training, policy support, and market incentives. At the same time, develop customized technology solutions to address the differences in production needs, resource endowment and technological capabilities of farmers in different regions and scales, such as designing lightweight and simplified green production technologies for small-scale farmers to reduce the cost and difficulty of application. Provide intelligent and precise technology solutions for large-scale farms to meet their efficient production needs. Through customized solutions, the implementation of green production practices in agriculture is promoted.
(4) Emphasize the long-term impact assessment of green production behaviors. Green production behaviors have far-reaching impacts on agricultural production, ecological environment, socio-economics and other fields, but their long-term impacts have not yet been adequately studied. Future research needs to systematically assess the long-term impacts of green production behaviors on ecosystem services such as soil health, water resource protection, and biodiversity through long-term field monitoring and ecological model simulation. At the same time, multi-dimensional assessment methods should be used to comprehensively analyze the long-term impacts of green production behaviors on economic and social benefits, such as farmers' income, competitiveness of agricultural products in the market, and rural social structure, so as to reveal their contribution to sustainable rural development.

## 5 Conclusions

This study conducted a systematic review of the research literature on green production behaviors of farmers in the last two decades by means of bibliometric analysis, focusing on its distribution capacity, research capacity, knowledge base and progress of the topic, research hotspots, evolutionary trends and quality distribution. This study adopts a combination of quantitative and qualitative methods and draws the following conclusions.

The green production practices of farmers have garnered significant attention within the international scholarly community, necessitating an interdisciplinary synthesis of knowledge that spans fields such as Environmental Science, Green and Sustainable Technology, and Environmental Studies. In many journals, “PSYCHOLOGY, EDUCATION, HEALTH” represents this field, and major publications include “JOURNAL OF CLEANER PRODUCTION”, “AGRICULTURAL SYSTEMS”, “LAND USE POLICY”, and “LAND USE POLICY”. These journals have significant academic authority and wide influence in the field.

In the realm of green production behavior among farmers, research activity is particularly pronounced in developing countries, with China emerging as a dominant contributor in terms of academic publications. USA has a high average citation rate, which indicates the depth and impact of its research. At the institutional level, CGIAR and NORTHWEST A F UNIVERSITY CHINA are in the lead. Concurrently, the Consultative Group on International Agricultural Research (CGIAR) has demonstrated exceptional research quality, as evidenced by its high average citation count. At the author level, Fu XH from China has the highest number of publications, while Adnan N from Saudi Arabia has the highest average citation count.

The co-citation analysis of references reveals that the foundational knowledge base of the field is predominantly categorized into three distinct domains: the refinement of theoretical models, the application of emerging technologies, and the development of research methodologies and evaluation frameworks. The professional literature focuses on the drivers of farmers' adoption of green production behaviors, theoretical model development, implementation evaluation, and application. The research theme has transitioned from the initial focus on theoretical elaboration and analysis of influencing factors to empirical investigations centered on individual determinants.

Keyword analysis shows that “adoption” and “green revolution” are the most frequent terms, while “attitudes” is a high-frequency and central relocation point. And “attitudes” are the most frequently occurring terms, while “attitudes” is the repositioning point with high frequency and centrality. The keyword cluster ring analysis shows that the research hotspots focus on the influencing factors of farmers' green production behaviors and the green revolution influencing farmers' agricultural technology adoption. Time-zone evolution mapping and emergent key analysis revealed the research evolution from the initial exploration of production technologies in agriculture to the attitudes and factors influencing the adoption of green production behaviors by farm households, in to the benefits of implementing green production behaviors. In addition, an analysis of the distribution of research quality shows that “planned behavior” has become a central theme, while “payments” and “aquaculture” point to future research directions.

Given the inherent complexity and dynamic instability of the literature ecosystem, the extraction of sufficiently reliable and valid data to quantitatively discern the overarching patterns within a specific research domain remains a formidable challenge. Bibliometric analysis is predicated upon the application of mathematical and statistical methodologies to systematically deconstruct and quantitatively assess the structural dimensions of scholarly literature within a defined area of research. It is an academic link closely related to scientific communication and grounded theory. Through the visualization and analysis of bibliometric, the research process, hotspots and trends of a specific research field can be clearly described. However, our study may have some limitations due to objective reasons. First, the research is predicated upon the Scientific Core Collection. While this collection is highly esteemed for its authoritative status and extensive coverage, its utilization may result in the omission of pertinent scholarly works that are cataloged in other significant repositories, including PubMed, Scopus, and Google Scholar. Second, the data were limited to articles and reviews, excluding literature types such as conference reports and books. Third, only English-language publications were selected for this study, which may have overlooked non-English publications such as Chinese, French, Spanish, etc. Nonetheless, we believe that the results of our analysis are sufficient to reflect the general state of affairs in the field of research on livelihood strategies of agricultural residents.

## Data Availability

The original contributions presented in the study are included in the article/supplementary material, further inquiries can be directed to the corresponding author.

## References

[1] Pfenning-Butterworth A Buckley LB Drake JM Farner JE Farrell MJ Gehman A-LM . Interconnecting global threats: climate change, biodiversity loss, and infectious diseases. Lancet Planetary Health. (2024) 8:e270–83. 10.1016/S2542-5196(24)00021-4 38580428 PMC11090248

[2] Liu M Chen X Jiao Y. Sustainable agriculture: theories, methods, practices and policies. Agriculture. (2024) 14:473. 10.3390/agriculture14030473

[3] Yuan X Li S Chen J Yu H Yang T Wang C . Impacts of global climate change on agricultural production: a comprehensive review. Agronomy. (2024) 14:1360. 10.3390/agronomy14071360

[4] Chen M Gao L Guo Z Dong Y Moallemi EA Xu Y . (2024). A cost-effective climate mitigation pathway for China with co-benefits for sustainability. Nat Commun. 15:1. 10.1038/s41467-024-53912-z 39488508 PMC11531567

[5] Hou C Chen H Long R. Research progress and prospect of green production: based on bibliometric analysis. Syst Eng Theory Pract. (2020) 40:2104–15. 10.12011/1000-6788-2019-2336-12

[6] Khalili NR Duecker S Ashton W Chavez F. From cleaner production to sustainable development: the role of academia. J Clean Prod. (2015) 96:30–43. 10.1016/j.jclepro.2014.01.09938253825

[7] Zhao P Gao Y Sun X. How does artificial intelligence affect green economic growth?-evidence from China. Sci Total Environ. (2022) 834:155306. 10.1016/j.scitotenv.2022.155306 35461946

[8] Li M Chen K. An empirical analysis of farmers' willingness and behavior in green agricultural production. J Huazhong Agricult Univer. (2020) 4:11–9. Available online at: http://hnxbw.cnjournals.net/hznydxsk/article/abstract/20200402?st=search28711241

[9] Loiseau E Saikku L Antikainen R Droste N Hansjürgens B Pitkänen K . Green economy and related concepts: an overview. J Clean Prod. (2016) 139:361–71. 10.1016/j.jclepro.2016.08.024

[10] Xu X Wang F Xu T Khan SU. How does capital endowment impact farmers' green production behavior? Perspectives on ecological cognition and environmental regulation. Land. (2023) 12:1611–1611. 10.3390/land12081611

[11] Wang D Lei M Xu X. Green production willingness and behavior: evidence from Shaanxi apple growers. Environ Dev Sustainab. (2024). 10.1007/s10668-024-04539-z

[12] Wang K Ma J. Agricultural socialized services and farmers' green production: research review and prospect. Agricult Econ Managem. (2024) 05:77–91. 10.3969/j.issn.1674-9189.2024.05.007

[13] van Eck NJ Waltman L. Software survey: vosviewer, a computer program for bibliometric mapping. Scientometrics. (2009) 84:523–38. 10.1007/s11192-009-0146-3 20585380 PMC2883932

[14] Zölch T Maderspacher J Wamsler C Pauleit S. Using green infrastructure for urban climate-proofing: an evaluation of heat mitigation measures at the micro-scale. Urban Forestry Urban Green. (2016) 20:305–16. 10.1016/j.ufug.2016.09.011

[15] Wei Y Song C Zhao Y Yang L Ma H. VISUAL econometric analysis of adaptive behavior of cliamte change and its enlightenment. Chin J Agricult Resour Regional Plann. (2024) 45:222–35. 10.7621/cjarrp.1005-9121.20240922

[16] Zhai J Sun X Hu X Tian J Huang Z. Evolutionary trends and hotspot analysis of livelihood strategy for agricultural residents based on bibliometrics. Agriculture. (2024) 14:1153–1153. 10.3390/agriculture14071153

[17] Chen C. Visualizing and exploring scientific literature with citespace. In: Proceedings of the 2018 Conference on Human Information Interaction&amp;Retrieval - CHIIR 18. New York, NY: Association for Computing Machinery (2018). p. 369–70. 10.1145/3176349.3176897

[18] Zhong Y Jiang X Yang Y Xu B Zhu Q Wang L . Visualization analysis of research hotspots on structural topology optimization based on citespace. Sci Rep. (2023) 13:1. 10.1038/s41598-023-45447-y 37875560 PMC10598221

[19] Chen C. CiteSpace II: detecting and visualizing emerging trends and transient patterns in scientific literature. J Am Soc Inform Sci Technol. (2005) 57:359–77. 10.1002/asi.20317

[20] Chen C Song I-Y Yuan X Zhang J. The thematic and citation landscape of data and knowledge engineering (1985–2007). Data Knowl Eng. (2008) 67:234–59. 10.1016/j.datak.2008.05.004

[21] Sun J Zhang D Peng S Wang Y Lin X. Insights of the fate of antibiotic resistance genes during organic solid wastes composting based on bibliometric analysis: development, hotspots, and trend directions. J Clean Prod. (2023) 425:138781. 10.1016/j.jclepro.2023.138781

[22] Kamrani P Dorsch I Stock WG. Do researchers know what the h-index is? And how do they estimate its importance? Scientometrics. (2021) 126:5489–508. 10.1007/s11192-021-03968-1

[23] Fernández-Cano A Torralbo M Vallejo M. Reconsidering price's model of scientific growth: an overview. Scientometrics. (2004) 61:301–21. 10.1023/B:SCIE.0000045112.11562.11

[24] Chen C Leydesdorff L. Patterns of connections and movements in dual-map overlays: a new method of publication portfolio analysis. J Assoc Inform Sci Technol. (2013) 65:334–51. 10.1002/asi.22968

[25] Shang X Liu Z Gong C Hu Z Wu Y Wang C. Knowledge mapping and evolution of research on older adults' technology acceptance: a bibliometric study from 2013 to 2023. Humanit Soc Sci Commun. (2024) 11:1. 10.1057/s41599-024-03658-2

[26] Chen K Chan AHS. Gerontechnology acceptance by elderly Hong Kong Chinese: a senior technology acceptance model (STAM). Ergonomics. (2014) 57:635–52. 10.1080/00140139.2014.895855 24655221

[27] Aldieri L Kotsemir M Vinci CP. The impact of research collaboration on academic performance: an empirical analysis for some european countries. Socioecon Plann Sci. (2018) 62:13–30. 10.1016/j.seps.2017.05.00327885969

[28] Zhang J Zhu L. Citation recommendation using semantic representation of cited papers' relations and content. Expert Syst Appl. (2022) 187:115826. 10.1016/j.eswa.2021.115826

[29] Baccini A Petrovich E. A global exploratory comparison of country self-citations 1996-2019. PLoS ONE. (2023) 18:e0294669. 10.1371/journal.pone.0294669 38157326 PMC10756561

[30] Hota PK Subramanian B Narayanamurthy G. Mapping the intellectual structure of social entrepreneurship research: a citation/co-citation analysis. J Business Ethics. (2019) 166:89–114. 10.1007/s10551-019-04129-4

[31] Li M Wang J Zhao P Chen K Wu L. Factors affecting the willingness of agricultural green production from the perspective of farmers' perceptions. Sci Total Environ. (2020) 738:140289–140289. 10.1016/j.scitotenv.2020.140289 32806378

[32] Xie H Huang Y. Influencing factors of farmers' adoption of pro-environmental agricultural technologies in China: meta-analysis. Land use policy. (2021) 109:105622. 10.1016/j.landusepol.2021.105622

[33] Mao H Zhou L Ying R Pan D. Time preferences and green agricultural technology adoption: field evidence from rice farmers in China. Land Use Policy. (2021) 109:105627–105627. 10.1016/j.landusepol.2021.105627

[34] Gao Y Zhao D Yu L Yang H. Influence of a new agricultural technology extension mode on farmers' technology adoption behavior in China. J Rural Stud. (2020) 76:173–83. 10.1016/j.jrurstud.2020.04.016

[35] Qing C Zhou W Song J Deng X Xu D. Impact of outsourced machinery services on farmers' green production behavior: evidence from chinese rice farmers. J Environ Manage. (2022) 327:116843. 10.1016/j.jenvman.2022.116843 36459784

[36] Niu Z Chen C Gao Y Wang Y Chen Y Zhao K. Peer effects, attention allocation and farmers' adoption of cleaner production technology: taking green control techniques as an example. J Clean Prod. (2022) 339:130700. 10.1016/j.jclepro.2022.130700

[37] Wang Y Zhu Y Zhang S Wang Y. What could promote farmers to replace chemical fertilizers with organic fertilizers? J Clean Prod. (2018) 199:882–90. 10.1016/j.jclepro.2018.07.222

[38] Adnan N Nordin SM Bahruddin MA Tareq AH. A state-of-the-art review on facilitating sustainable agriculture through green fertilizer technology adoption: assessing farmers behavior. Trends Food Sci Technol. (2019) 86:439–52. 10.1016/j.tifs.2019.02.040

[39] Chen C. Science mapping: a systematic review of the literature. J Data Inform Sci. (2017) 2:1–40. 10.1515/jdis-2017-0006

[40] Gao Y Zhang X Lu J Wu L Yin S. Adoption behavior of green control techniques by family farms in China: evidence from 676 family farms in Huang-Huai-Hai plain. Crop Prot. (2017) 99:76–84. 10.1016/j.cropro.2017.05.012

[41] Zhang L Li X Yu J Yao X. Toward cleaner production: what drives farmers to adopt eco-friendly agricultural production? J Clean Prod. (2018) 184:550–8. 10.1016/j.jclepro.2018.02.272

[42] Zhao L Wang C Gu H Yue C. Market incentive, government regulation and the behavior of pesticide application of vegetable farmers in China. Food Control. (2018) 85:308–17. 10.1016/j.foodcont.2017.09.016

[43] Liu D Zhu X Wang Y. China's agricultural green total factor productivity based on carbon emission: an analysis of evolution trend and influencing factors. J Clean Prod. (2021) 278:123692. 10.1016/j.jclepro.2020.123692

[44] Huang R Yan P Yang X. Knowledge map visualization of technology hotspots and development trends in China's textile manufacturing industry. IET Collab IntelligManufact. (2021) 3:243–51. 10.1049/cim2.12024

[45] Seop PJ Kim NR Han E-J. Analysis of trends in science and technology using keyword network analysis. J Korea Soc Indust Inform Syst. (2018) 23:63–73. 10.9723/jksiis.2018.23.2.063

[46] Pinstrup-Andersen P Hazell PBR. The impact of the green revolution and prospects for the future. Food Rev Int. (1985) 1:1–25. 10.1080/87559128509540765

[47] Pesta B Fuerst J Kirkegaard E. Bibliometric keyword analysis across seventeen years (2000–2016) of intelligence articles. J Intellig. (2018) 6:46. 10.3390/jintelligence6040046 31162473 PMC6480778

[48] Cheng Q Wang J Lu W Huang Y Bu Y. Keyword-citation-keyword network: a new perspective of discipline knowledge structure analysis. Scientometrics. (2020) 124:1923–43. 10.1007/s11192-020-03576-5

[49] Pimentel D. Green revolution agriculture and chemical hazards. Sci Total Environ. (1996) 188:S86–98. 10.1016/0048-9697(96)05280-1 8966546

[50] Niazi T. Rural poverty and the green revolution: the lessons from pakistan. J Peasant Stud. (2004) 31:242–60. 10.1080/0306615042000224294

[51] Wang X Ma Y Li H Xue C. How does risk management improve farmers' green production level? Organic fertilizer as an example. Front Environm Sci. (2022) 10. 10.3389/fenvs.2022.946855

[52] Liu W Arshad MU Zhang L Wei J Fu Y. Uncovering the key factors influencing sustainable green production behavior among chinese medicinal herb growers. Heliyon. (2023) 9:e22385. 10.1016/j.heliyon.2023.e22385 38034667 PMC10687229

[53] Hou D Wang X. Inhibition or promotion?–the effect of agricultural insurance on agricultural green development. Front Public Health. (2022) 10:910534. 10.3389/fpubh.2022.910534 35937251 PMC9352884

[54] Bauin S Michelet B Schweighoffer MG Vermeulin P. Using bibliometrics in strategic analysis: “understanding chemical reactions” at the cnrs. Scientometrics. (1991) 22:113–37. 10.1007/BF02019278

[55] Callon M Courtial JP Laville F. Co-word analysis as a tool for describing the network of interactions between basic and technological research: the case of polymer chemsitry. Scientometrics. (1991) 22:155–205. 10.1007/BF02019280

[56] Shen J Zhu Q Hou Y Cong W-F Xu W Xu J . Agriculture green development in China: insights and advances. Front Agricult Sci Eng. (2024) 11:5–19. 10.15302/J-FASE-2024535

[57] Xianyu Y Long H Wang Z Meng L Duan F. The impact of tea farmers' cognition on green production behavior in jingmai mountain: chain mediation by social and personal norms and the moderating role of government regulation. Sustainability. (2024) 16:8885–8885. 10.3390/su16208885

[58] Xu Z Meng W Li S Chen J Wang C. Driving factors of farmers' green agricultural production behaviors in the multi-ethnic region in China based on NAM-TPB models. Global Ecol Conserv. (2024) 50:e02812–e02812. 10.1016/j.gecco.2024.e02812

[59] Dai Q Cheng K. What drives the adoption of agricultural green production technologies? An extension of tam in agriculture. Sustainability. (2022) 14:14457. 10.3390/su142114457

[60] He Z Jia Y Ji Y. Analysis of influencing factors and mechanism of farmers' green production behaviors in China. Int J Environ Res Public Health. (2023) 20:961–961. 10.3390/ijerph20020961 36673714 PMC9859079

[61] Liu Y Yang J Zhang G Cui X. Driving factors of green production behaviour among farmers of different scales: evidence from North China. Agricult. Econ. (2024) 70:474–94. 10.17221/188/2024-AGRICECON

[62] Lin X Zhou R Liang D Xia L Zeng L Chen X. The role of microbiota in autism spectrum disorder: a bibliometric analysis based on original articles. Front Psychiatry. (2022) 13. 10.3389/fpsyt.2022.976827 36172516 PMC9512137

[63] Yang C Liang X Xue Y Zhang Y Xue Y. Can government regulation weak the gap between green production intention and behavior? based on the perspective of farmers' perceptions. J Clean Prod. (2023) 434:139743. 10.1016/j.jclepro.2023.139743

[64] Zhang M Zhou L Zhang Y Zhou W. Economic and environmental effects of farmers' green production behaviors: evidence from major rice-producing areas in Jiangxi province, China. Land. (2024) 13:1668–1668. 10.3390/land13101668

[65] Adnan N Nordin SM bin Abu Bakar Z. Understanding and facilitating sustainable agricultural practice: a comprehensive analysis of adoption behaviour among malaysian paddy farmers. Land Use Policy. (2017) 68:372–82. 10.1016/j.landusepol.2017.07.046

[66] Adnan N Nordin SM Rahman I Noor A. Adoption of green fertilizer technology among paddy farmers: a possible solution for malaysian food security. Land Use Policy. (2017) 63:38–52. 10.1016/j.landusepol.2017.01.022

[67] Liu Y Sun D Wang H Wang X Yu G Zhao X. An evaluation of China's agricultural green production: 1978–2017. J Clean Prod. (2019) 243:118483–118483. 10.1016/j.jclepro.2019.118483

[68] Liu Y Ruiz-Menjivar J Zhang L Zhang J Swisher ME. Technical training and rice farmers' adoption of low-carbon management practices: the case of soil testing and formulated fertilization technologies in Hubei, China. J Clean Prod. (2019) 226:454–62. 10.1016/j.jclepro.2019.04.026

[69] Zhang L Li Y Li Q. A graph-based keyword extraction method for academic literature knowledge graph construction. Mathematics. (2024) 12:1349. 10.3390/math12091349

